# Jointly estimating individual and group networks from fMRI data

**DOI:** 10.1162/netn_a_00457

**Published:** 2025-07-29

**Authors:** Don van den Bergh, Linda Douw, Zarah van der Pal, Tessa F. Blanken, Anouk Schrantee, Maarten Marsman

**Affiliations:** Department of Psychological Methods, University of Amsterdam; Department of Anatomy and Neurosciences, Amsterdam UMC location Vrije Universiteit Amsterdam; Department of Radiology and Nuclear Medicine, Amsterdam UMC location University of Amsterdam; Department of Clinical Psychology, University of Amsterdam

**Keywords:** Multilevel model, Bayesian inference, Resting-state fMRI, Gaussian graphical model, Curie-Weiss model

## Abstract

In fMRI research, graphical models are used to uncover complex patterns of relationships between brain regions. Connectivity-based fMRI studies typically analyze nested data; raw observations, for example, BOLD responses, are nested within participants, which are nested within populations, for example, healthy controls. Often, studies ignore the nested structure and analyze participants either individually or in aggregate. This overlooks the distinction between within-participant and between-participant variance, which can lead to poor generalizability of results because group-level effects do not necessarily reflect effects for each member of the group and, at worst, risk paradoxical results where group-level effects are opposite to individual-level effects (e.g., Kievit, Frankenhuis, Waldorp, & Borsboom, 2013; Robinson, 2009; Simpson, 1951). To address these concerns, we propose a multilevel approach to model the fMRI networks, using a Gaussian graphical model at the individual level and a Curie-Weiss graphical model at the group level. Simulations show that our method outperforms individual or aggregate analysis in edge retrieval. We apply the proposed multilevel approach to resting-state fMRI data of 724 healthy participants, examining both their commonalities and individual differences. We not only recover the seven previously found resting-state networks at the group level but also observe considerable heterogeneity in the individual-level networks. Finally, we discuss the necessity of a multilevel approach, additional challenges, and possible future extensions.

## INTRODUCTION

The brain functions as a network (Bassett & Sporns, 2017). Functional connectivity assessed by activity patterns from different areas of the brain reveals both group-level organizational aspects, such as the robust presence of the default mode network (Buckner & DiNicola, 2019; Gratton et al., 2018), as well as individual differences in the general or ‘healthy’ functional network (e.g., Xiao et al., 2023). Many studies first extract a network for each participant and then relate topological properties to behavioral measures. However, it is becoming increasingly clear that there is a large shared functional network topology across individuals, with individual differences being smaller (Gratton et al., 2018) but consistent across groups of individuals (Gordon, Laumann, Adeyemo, & Petersen, 2017; Mueller et al., 2013; Seitzman et al., 2019). This underscores the need to better understand functional brain networks at the group level, as a clearer grasp of the group level can facilitate identifying and studying different subgroups.

Graphical models have become an integral part of modeling functional connectivity using fMRI data. Such data typically consist of repeated measurements per participant, even when participants undergo a single scan, and to analyze these data, a separate network is estimated for each participant. However, while conducting their analyses at the **individual level**, researchers often wish to make inferences at the **group level** rather than the individual level. For example, studies that focus on resting-state fMRI in patients with major depression clearly intend to generalize over patients with major depression (e.g., see Dichter, Gibbs, & Smoski, 2015; Wang, 2012). For such group-level inferences, it is critical to separate individual differences in functional connectivity from population-level patterns to draw meaningful conclusions. Note that this is not specific to fMRI but holds, in general, whenever researchers aim to generalize findings from individual-level data to broader populations (see, e.g., Mangalam & Kelty-Stephen, 2021).

In general, fMRI data have a natural nested structure. Within each participant, the raw observations (i.e., the BOLD responses) form a time series that contains within-subject variance. Between participants, these individual time series collectively contain between-subjects variance. However, fMRI studies tend to neglect this nested structure in their data, in part because available network analysis software cannot account for it. In practice, two distinct approaches that involve different forms of aggregation are used: (a) Researchers analyze participants individually and then aggregate their results for further inference, or (b) researchers first aggregate the person-level data, analyze the aggregated data, and use the results for further inference (e.g., see Cho, Korchmaros, Vogelstein, Milham, & Xu, 2021). Both approaches have drawbacks. While the first approach ignores the shared group-level information among participants, the second approach assumes that there are no meaningful individual differences. When analyzed in these two ways, researchers cannot make full use of the available fMRI data and, therefore, fail to distinguish between within-person and between-person variance (Pollet, Stulp, Henzi, & Barrett, 2015; Schad, Nicenboim, & Vasishth, 2024; Watkins & Martire, 2015).

The main drawback of aggregation is that it ignores individual heterogeneity. This can result in incorrect standard errors because the aggregation reduces the observed variability, underestimating the true uncertainty in the data (Krull & MacKinnon, 2001). Consequently, inferences based on aggregated data may be biased, leading to misleading conclusions about relationships or effects. In the most extreme case, ignoring individual heterogeneity may lead to **Simpson’s paradox**, where the relationship at the individual level does not have to be the same as the relationship at the group level. While Simpson’s paradox has not been widely reported in the literature, it has been found in a number of other studies. For example, Roberts, Hach, Tippett, and Addis (2016) observed that some correlations between brain regions flip signs depending on whether they are analyzed individually or in aggregate. In another study, Zimmermann et al. (2024) first associated neuronal activity with functional connectivity at the group level, finding a positive correlation in both the average healthy and the average brain cancer cohorts. However, performing the same analysis at the individual level revealed much more variance and an overall negative correlation in the patient population, a prime example of Simpson’s paradox. Thus, analyzing participants individually or in aggregate may compromise both the generalizability and the direction of results, as they may be reversed depending on whether analyses are performed at the participant level or at the group level.

To take full advantage of fMRI data in network analysis, it is necessary to model its nested structure using a multilevel graphical model that is sensitive to both within-participant and between-participant variation. Such a multilevel approach has the advantage of pooling information across participants to improve the precision of individual network estimates (Chiquet, Grandvalet, & Ambroise, 2011; Peterson, Stingo, & Vannucci, 2015). Several approaches have been proposed to jointly model individual-level and group-level networks (e.g., see Peterson & Stingo, 2021; Tsai, Koyejo, & Kolar, 2022), but these are not widely used in connectivity-based fMRI network research (e.g., see van der Pal et al., 2024).

To overcome the drawbacks of aggregating, we simultaneously model individual level and group level with a Bayesian **multilevel model**. Our approach yields explicit evidence for the presence or absence of edges in both the individual-level and the group-level network. This helps to inform practical decisions about the structure of the group-level network and its specific connections, and it provides a solid foundation for building theory about the global structure of functional connectivity. At the same time, we can investigate individual differences and deviations from the group-level structure.

The remainder of this paper is organized as follows. In the next section, we present the general setup of our Bayesian multilevel model. We introduce the **Gaussian graphical model** (GGM) to describe the individual-level networks. Next, we describe the **Curie-Weiss model** and how we use it as a group-level network to capture the commonalities among the participants. We then compare the performance of our proposed multilevel model against analyzing participants individually or in aggregate using simulated data. Afterward, we illustrate the multilevel model by applying it to a resting-state fMRI dataset consisting of 724 participants with 956 time points per participant (Tijhuis et al., 2022), demonstrating the concrete benefits outlined above. From the models considered here, we can obtain probabilities for the inclusion and exclusion of edges at both the individual-level and group-level networks. We use the inclusion probabilities to compare model performance using receiver operating characteristic (ROC) curves. Finally, we discuss the need for multilevel modeling, the limitations of the chosen individual- and group-level models, and possible ways to further extend our approach.

## MATERIALS AND METHODS

### A Bayesian Multilevel Model for Functional Connectivity

The key idea of our approach is to formulate the joint distribution of the group-level network G and the individual-level networks $S_{n}$ for participants *n* = 1, …, *N* as follows:

$$\underset{\text{Joint Distribution}}{\underbrace{p\left(G,S_{1},\ldots ,S_{N}|\text{data}\right)}}\propto \underset{\text{Individual Level}}{\underbrace{\prod_{n=1}^{N}\;p\left({\text{data}}_{n}|S_{n}\right)}}\;\underset{\text{Group Level}}{\underbrace{p\left(S_{n}|G\right)p\left(G\right)}}.$$ (1)

**Figure 1.**
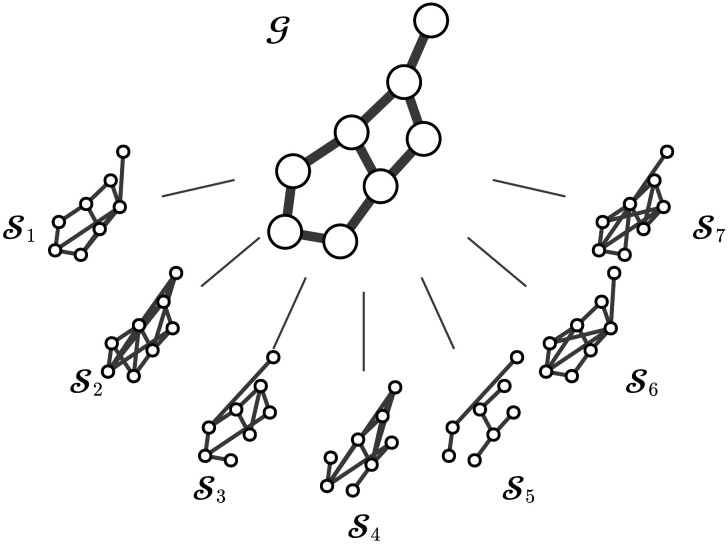
An illustrative visualization of a group-level network G and individual-level networks S for seven participants and eight nodes.

This formulation has three important implications. First, it formalizes the nested structure of fMRI data; the raw observations of participant *n* (data*_n_*) are nested within the individual-level network ($S_{n}$), and the individual-level networks are nested within the group (G). Second, it implies that the group-level network explains all the commonalities between the individual-level networks. Third, it provides a flexible framework for specifying individual-level and group-level models separately. Apart from these three properties mentioned above, there are no constraints on the type of individual-level or group-level model. Figure 1 visualizes the decomposition in Equation 1.

The next sections introduce conceptually our choices for the individual-level and group-level models. Note, however, that as long as the three properties outlined above are satisfied, one could, in principle, mix and match any individual-level or group-level model.

### The Individual-Level Network

Given the decomposition in Equation 1, the first challenge is to specify an individual-level network model. Here, we use the GGM (Lauritzen, 1996; Uhler, 2018). The GGM is often used to uncover the network structure of continuous data and assumes that the data follow a multivariate normal distribution. Specifically, we assume that the (preprocessed) signal *x*_*tn*_ of participant *n* at time point *t* is normally distributed with mean zero and an unknown covariance matrix Σ. We are primarily interested in the conditional (in)dependence relations between the nodes, that is, the predictive value of one brain region on another after controlling for all other regions. The conditional dependence relations are captured by partial correlations, and a partial correlation of 0 indicates that two regions are conditionally independent, that is, any correlation between them can be fully explained by accounting for the activity in the rest of the network. Conveniently, the partial correlations can be obtained by standardizing the inverse of the covariance matrix, the precision matrix Ω. A zero off-diagonal element in the precision matrix implies that two variables are conditionally independent (Dempster, 1972).

We adopt a Bayesian approach to learn the parameters of the GGM and next focus on the likelihood and prior distributions. Consider the data for one participant, denoted as ***X**_n_*, with *Τ_n_* observations on *p* nodes. The subscript *n* indicates the participant and ranges from 1 to *N*. To ease the notation, we further suppress the participant subscript when describing the GGM. The likelihood of the GGM for a single participant with sample covariance matrix ***S*** and *T* observations is given by

$$L\left(X|\Omega \right)=\prod_{t=1}^{T}N\left(X_{t}|0,\Omega^{-1}\right),$$

$$={\left(2\pi \right)}^{-\frac{T}{2}}\det{\left(\Omega \right)}^{\frac{T}{2}}\exp\left(-\frac{1}{2}\sum_{t=1}^{T}X_{t}^{T}\Omega X_{t}\right),$$

$$={\left(2\pi \right)}^{-\frac{T}{2}}\det{\left(\Omega \right)}^{\frac{T}{2}}\exp\left(-\frac{T}{2}Tr\left(\Omega S\right)\right),$$

where the last line make use of the cyclic and linear properties of the trace, that is, $\sum_{t=1}^{T}X_{t}^{T}\Omega X_{t}=\sum_{t=1}^{T}Tr\left(X_{t}^{T}\Omega X_{t}\right)=\sum_{t=1}^{T}Tr\left(X_{t}X_{t}^{T}\Omega \right)$.

There are many approaches to estimate the parameters of the GGM (e.g., see Vogels, Mohammadi, Schoonhoven, & Birbil, 2024). Here, we use the spike-and-slab approach proposed by Wang (2015). Specifically, we adopt the following prior distribution over the precision matrix:

$$p\left(\Omega \right)\propto \prod_{p=1}^{P}p\left(\omega_{p}\right)\prod_{p^{\prime }<p}p\left(\omega_{p^{\prime }p}\right).$$

The normalizing constant of the prior is intentionally omitted because there is never a need to compute it. For the elements on the diagonal of the precision matrix, that is, *ω_pp_*, we use an exponential distribution with hyperparameter *λ*, which is defined as *p*(*ω_pp_*) = *λ* exp (−*ω_pp_λ*). For the off-diagonal elements of the precision matrix, we use a spike and slab prior, $p\left(\omega_{p^{\prime }p}\right)=N\left(\omega_{p^{\prime }p}|0,{\left(v_{1}^{\gamma_{p^{\prime }p}}v_{0}^{1-\gamma_{p^{\prime }p}}\right)}^{2}\right)$, where the two variances $v_{0}^{2}$ and $v_{1}^{2}$ are hyperparameters and need to be specified. Here, we assume that *v*_0_ < < *v*_1_, such that the prior variance $v_{0}^{2}$ implies a very narrow distribution about zero and the off-diagonal element *ω*_*p*′*p*_ is shrunk to a value close to zero. Conversely, $v_{1}^{2}$ is specified such that the implied prior density is very wide and hardly shrinks *ω*_*p*′*p*_ at all. The variable γ is an indicator that indicate which variance component is used in the prior; when it is equal to 1, the prior variance is equal to $v_{1}^{2}$; otherwise, when the indicator is 0, the prior variance is $v_{0}^{2}$. Thus, the spike-slab-approach model implies that if γ_*p*′*p*_ = 0, then *ω*_*p*′*p*_ ≈ 0 is close to 0 and nodes *p*′ and *p* are approximately conditionally independent.

### The Group-Level Network

The second part of Equation 1 concerns the group-level model, for which we use the Curie-Weiss model. Originally developed in physics to describe magnetism (see, e.g., Kac, 1968), the Curie-Weiss model is now used more broadly, for example, in psychometrics (Marsman, Tanis, Bechger, & Waldorp, 2019). In general, the Curie-Weiss model defines a probability distribution over binary variables that is characterized by the main effects of the variables and the average interaction between them. Here, we use the Curie-Weiss model to describe the edges that are present (1) or absent (0) in each participant’s network; the indicator variables *γ* in the spike-and-slab model for the individual-level networks. Specifically, we denote the edges in the network of participant *n* by *γ_n_* (i.e., the lower triangle of the adjacency matrix), use the subscript *e* = 1, …, *Ε* to denote an edge (*e* is shorthand for *p*′*p* in the previous section), and define the Curie-Weiss model as

$$p\left(\gamma_{n}|\mu ,\sigma \right)=Z{\left(\mu ,\sigma \right)}^{-1}\exp\left(\sum_{e=1}^{E}\gamma_{en}\mu_{e}+\frac{\sigma }{e}{\left(\gamma_{n}^{+}\right)}^{2}\right).$$

Here, *μ* denotes the vector of main effects and *σ* denotes the average interaction among the edges. The normalizing constant *Z*(*μ*, *σ*) is given by the sum over all possible states of *γ_n_*, that is, all possible configurations of edges being either present or absent in the group network. The term ${\left(\gamma_{n}^{+}\right)}^{2}$ is the square of the number of edges in the network of participant *n* and describes the interactions between the edges. This term is obtained by multiplying all pairs of edges, that is, ${\left(\gamma_{n}^{+}\right)}^{2}=\sum_{e,e^{\prime }}^{E}\gamma_{ek}\gamma_{e^{\prime }k}={\left(\sum_{e=1}^{E}\gamma_{en}\right)}^{2}$. Note that when *σ* = 0, the Curie-Weiss model reduces to a product of independent Bernoulli distributions, where the edges are independent and *μ_e_* is the logit of the probability that edge *e* is included in the individual-level networks. This interpretation still holds for nonzero *σ*, that is, a larger value for *μ_e_* increases the probability that edge *e* is included, although the relationship with the probability becomes more complex. The interaction parameter can be interpreted as the tendency of the edges to be in the same state.

### Implementation

We use a Bayesian approach to fit the multilevel model outlined above. To this end, we have derived a **Gibbs sampler** for the individual-level model and group-level model; for technical derivations, see Appendix B in the Supporting Information. A Gibbs sampler is particularly well suited to multilevel applications because it allows one to separate the updating of the individual-level model from the updating of the group-level model. In addition, a pragmatic advantage of our choice of group-level model is that the individual-level networks are independent conditioning on the group level. That is, the group network explains all cohesion between the individual networks. This allows for parallelizing the update of the individual-level models. We assessed the convergence of the Markov chain Monte Carlo (MCMC) chains by computing the $\hat{R}$-statistics for all parameters of the Curie-Weiss model and ensured that these were all below 1.05 (Vehtari, Gelman, Simpson, Carpenter, & Bürkner, 2021). All analyses were implemented in the Julia programming language (Bezanson, Edelman, Karpinski, & Shah, 2017). One motivation for using Julia is that the posterior samples for the individual-level graph structure could be efficiently represented by the so-called BitArrays, a type that represents each binary value with a single bit. In the empirical example presented later, with 116 nodes and 724 participants, this means that to store the adjacency matrices for all individual-level networks for a single MCMC iteration, only 1.1 megabytes are needed instead of 74.3 megabytes, as would be the case if we had used an array of 64-bit integers. All code and analyses are available at https://github.com/vandenman/supplementJointlyEstimating.

## NUMERICAL ILLUSTRATIONS

This section demonstrates the benefits of a multilevel approach over analyzing participants individually or in the aggregate using simulated data. We focus on two simulations, the first showcases the ability of the multilevel model to pool information across participants, resulting in increased power when the participants are somewhat homogenous. The second simulation illustrates the capacity of the multilevel model to detect model misspecification, for example, when there are multiple groups but the model assumes that there is only one. Appendix C in the Supporting Information provides an additional simulation that illustrates how well our approach can recover data simulated with a modular structure. Appendix D in the Supporting Information showcases the impact of temporal autocorrelation on the parameter estimates of the GGM.

We used the multilevel model to simulate the data. This means that we first simulated a true group-level network from which true individual-level networks were drawn. The individual-level networks were subsequently used to simulate true precision matrices, which were drawn from a *G*-Wishart distribution (Lenkoski, 2013; Roverato, 2002). Finally, the simulated individual-level precision matrices were used to simulate observations from a multivariate normal distribution, representing the raw time series.

In the first simulation, we simulated data for *T* time points with *T* ∈ 50, 100, 500, and 1,000, and *N* participants *N* ∈ 50, 100, 300, and 500 with *P* = 40 nodes. The group-level network was simulated with characteristics similar to those observed in the data example presented in the next section. That is, in the estimated group network, about 16% of all possible edges were present, and thus, we simulated the group network with approximately the same percentage of edges. Next, we simulated values for the *μ* parameter of the Curie-Weiss model. The parameter values were informed by the parameter estimates of the data example later on to ensure that the generated datasets are realistic. If an edge was included in the newly simulated group network, we drew *μ* from a positive half-normal distribution, $N^{+}\left({\bar{\mu }}^{+},\sigma_{\mu^{+}}\right)$, where ${\bar{\mu }}^{+}=2.73$ and $\sigma_{\mu^{+}}=2.33$, the posterior mean and standard deviation for positive values of *μ* in the data example. If an edge was excluded from the newly simulated group network, we drew *μ* from a negative half-normal distribution, $N^{-}\left({\bar{\mu }}^{-},\sigma_{\mu^{-}}\right)$, where ${\bar{\mu }}^{-}=3.16$ and $\sigma_{\mu^{+}}=3.35$, the posterior mean and standard deviation for negative values of *μ* in the data example. In addition, we varied a percentage *ρ* across simulations with *ρ* ∈ 0, 10, 30, 50, and 100, such that $\mu^{+}=3.16p{/}_{100}$ and $\sigma_{\mu^{+}}=3.35p{/}_{100}$. This implies that if *ρ* = 0, there is no group network and all simulated participants are independent, and that if *ρ* = 50, the dependency is half as strong as was found in the data example. In principle, *ρ* can be interpreted as the homogeneity among the individuals, albeit that for *ρ* = 100, individuals do not become identical but rather equally similar as estimated in the data example. The *σ* parameter of the Curie-Weiss model was set to 0.1. Each unique combination for the time points *T*, the number of participants *N*, and the proportion *ρ* was repeated five times.

Next, we analyze each simulated dataset in three ways. The first analysis uses our proposed Bayesian multilevel model. The second analysis estimates the results for each participant individually. Note that estimating a network for each participant individually (implicitly) assumes that participants are independent and that there is no common structure, corresponding to the condition where *ρ* = 0. The third analysis aggregates the raw data before analyzing it, essentially assuming that participants are exchangeable and that there are no (meaningful) individual differences. In the third analysis, we aggregated the data by first computing the sample precision matrices for each simulated participant and then averaging them. While this is not a common approach to aggregating (e.g., see Cho et al., 2021), we did so because the individual-level model we used (the GGM) estimates the precision matrix and not the covariance matrix. Therefore, it is more appropriate to average the sample precision matrices, rather than the sample covariance matrices.

**Figure 2.**
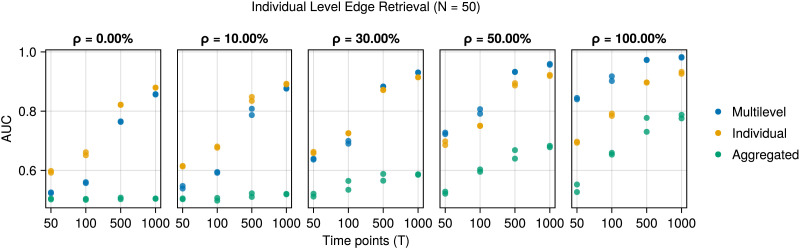
AUC for edge retrieval of the individual-level networks (*y*-axis) against the number of time points (*x*-axis) for the three methods on simulated data (colors and shapes). Across the columns, the cohesion among the participants increases. When the homogeneity *ρ* is larger than 30, the multilevel model outperforms the analysis of the individual participants, which, in turn, outperforms the analysis of the aggregated data. If the cohesion is 0, then the multilevel and individual methods perform similarly.

First, we examine the recovery of the edges in the individual-level networks by looking at the **area under the curve (AUC)** of the ROC curves (Fawcett, 2006). Each method uses the posterior inclusion probability to predict the presence of an edge for each individual. For the multilevel and individual methods, these probabilities differ across edges and participants, while for the aggregated method, the inclusion probability differs only across edges, as this method assumes no meaningful differences between participants and estimates a single model. Figure 2 shows the result for *N* = 50; the other results are comparable and are shown in Appendix A in the Supporting Information.

The multilevel model’s performance increases with the individuals’ homogeneity and seemingly performs best out of the three methods from *ρ* = 30% onward. At *ρ* = 30%, around 25% of the simulated participants have an edge in their individual-level networks that is absent in the group networks. Conversely, about 28% misses an edge that is present in the group network. Note that as *ρ* increases, participants become more similar, but are by no means identical, as indicated by the poor performance of the aggregated method.

**Figure 3.**
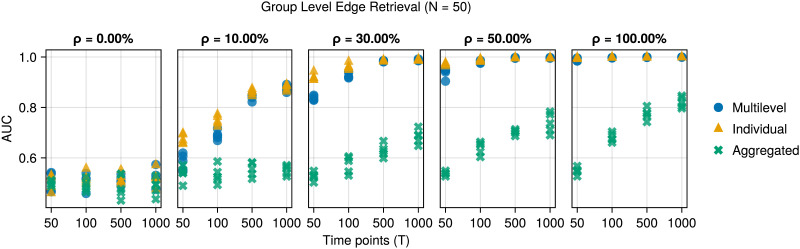
AUC for edge retrieval of the group-level network (*y*-axis) against the number of time points (*x*-axis) for the three methods on simulated data (colors and shapes). Across columns, the cohesion increases. The multilevel and individual methods perform similarly, while the aggregated method performs poorly.

Next, we examine the recovery of the edges in the group-level network. Figure 3 shows the recovery of the edges across the group-level networks by examining the AUC.

Here, the pattern is similar. If the participants are relatively heterogeneous (*ρ* ≤ 30%), then the individual method outperforms the multilevel method. However, as the number of time points or the homogeneity increases, the performance of the multilevel and individual methods becomes indistinguishable. The aggregated method performs poorly.

Figures 2 and 3 demonstrate the ability of the multilevel model to pool information across individuals. If the participants are somewhat homogeneous (*ρ* > 30%), this leads to improved edge retrieval at the individual level, especially when there are few time points per participant.

In a second simulation, we illustrate how to quantify model misfit at the group level by analyzing three simulated datasets (all *T* = 1,000, *P* = 60, and *N* = 100). In the first dataset, the data are simulated as before, but the variance between the individual-level networks is low, that is, the participants are homogeneous. In the second dataset, the data are simulated as before, but the variance between the individual-level networks is high, that is, the participants are heterogeneous. This variance is manipulated by adjusting the *μ* in the Curie-Weiss model; a large absolute value *μ_e_* for edge *e* leads to low variance in that edge across the participants. In the third dataset, the data are simulated from a mixture of two group networks but analyzed with a group model that ignores this subgroup structure. The purpose of this simulation is to show what happens when the group-level network is misspecified. We randomly generate one group-level network like before. The second group-level network is obtained by flipping 20% of the edges in the first group-level network. Next, we simulated 80 participants using the first group network and 20 participants using the second group network. Note that in this third dataset, unlike the first two datasets, it is not possible for a single group-level network to accurately describe the data and, thus, the model is misspecified.

**Figure 4.**
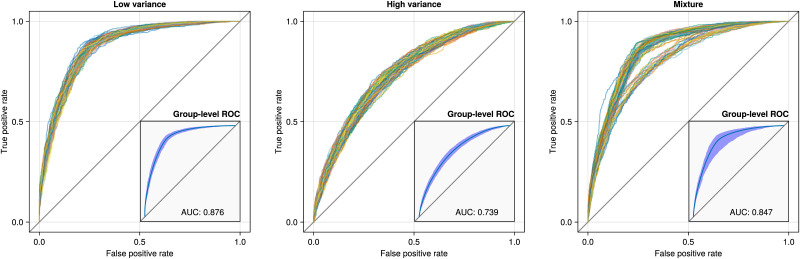
ROC curves predicting the individual-level networks using the group-level inclusion probabilities. The inset plots show the average ROC curve with a 95% confidence band and the corresponding AUC.

After analyzing the datasets, we use the estimated group-level network to predict the edges in the estimated individual-level network. First, we determine which edges to include in the individual networks using a threshold for the posterior inclusion probabilities of a value of 0.5. We chose a threshold of 0.5 because it balances the trade-off between sensitivity and specificity, providing a reasonable criterion for edge inclusion. Next, we use the group-level edge inclusion probabilities to predict the thresholded individual-level network structures. We use ROC curves to assess how well the group-level network describes the estimated individual-level network of each simulated participant. Figure 4 shows these ROC curves for all three simulated datasets.

The left and middle panels show the difference between a homogeneous and heterogeneous group of participants, respectively. As the variance in the networks at the individual level increases, the AUC decreases, which makes sense because a higher variance implies that individuals are more different from the group level. In the rightmost panel, the model is misspecified because the data were simulated from two group-level networks. This is evident from the ROC curves, as there are two distinct bundles of ROC curves. However, at the aggregate level, as shown in the inset plot of the rightmost panel, the misfit becomes difficult to see and cannot be determined solely from the AUC or average ROC curve. The varying width of the 95% confidence band does suggest misfit but note that this confidence band can only be computed using the individual-level ROC curves. Thus, any approach based on individual analyses or aggregated analyses would fail to detect this misfit. Note that the high AUC in the rightmost panel may give an impression of a good model fit, but in reality, the results would generalize poorly to the population belonging to the second group network.

In the simulations, we used the median probability model to transform the inclusion probabilities for the individual-level network to binary edges (Barbieri & Berger, 2004), which implies that only edges with an inclusion probability above 0.5 were retained. Given our model specification, this threshold is equivalent to an inclusion Bayes factor of 1, because the prior edge inclusion probabilities are 0.5, so an edge is present if it is more likely to be included than not. The inclusion Bayes factor quantifies the change from prior to posterior inclusion probability and is defined as (Sekulovski et al., 2024),

$${BF}_{e}=\underset{\text{Posterior inclusion odds}}{\underbrace{\frac{p\left(\gamma_{e}=1|\text{data}\right)}{p\left(\gamma_{e}=0|\text{data}\right)}}}/\underset{\text{Prior inclusion odds}}{\underbrace{\frac{p\left(g_{e}=1\right)}{p\left(\gamma_{e}=0\right)}}}$$ (2)

When analyzing empirical data, it is common to use a more conservative threshold based on the inclusion Bayes factor, such as 3 or 10 (Kass & Raftery, 1995; Van Doorn et al., 2021). The threshold then leads to three categories of evidence: evidence for edge inclusion (BF*_e_* ≥ 3), evidence for edge exclusion (BF*_e_* ≤ 1/3), and edges for which there is insufficient evidence for a meaningful conclusion (1/3 < BF*_e_* < 3).

## EMPIRICAL EXAMPLE

This section presents the results of the multilevel model on the resting-state fMRI data from the Lifespan Human Connectome Project Release 2.0 from February 2021. We focus on the group-level structure and individual differences.

### fMRI Dataset

Here, we illustrate the method on data from 724 healthy participants, whose data are available to the public at https://db.humanconnectome.org. The scanning protocols for these data have been published before (Harms et al., 2018). In short, participants underwent two runs of resting-state fMRI, with 488 volumes being collected per run (total time of 26 min). Details on the processing of data and connectivity calculation can be found on GitHub (Tijhuis et al., 2023). The connectivity matrices we used can be obtained from Tijhuis et al. (2022). We used the Schaefer 100 atlas (Schaefer et al., 2018) in combination with the 16 subcortical areas of the scale I atlas (Tian, Margulies, Breakspear, & Zalesky, 2020) and organized the nodes according to the seven resting-state networks defined by Yeo et al. (2011). After preprocessing, the dataset consisted of an fMRI time series of 116 nodes at 956 time points for each participant.

### The Group-Level Model

**Figure 5.**
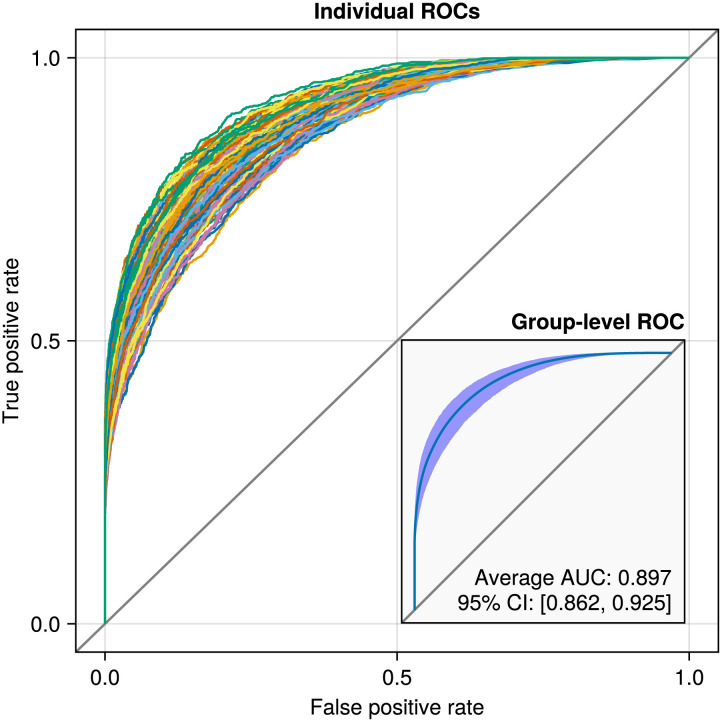
ROC curves for all participants (main plot) and averaged across participants with a 95% confidence band (inset plot). Evident is that the group-level network adequately describes the individual-level networks.

Here, we interpret the group-level results from the multilevel model. However, before interpreting the group-level network, it is a good idea to assess the model fit, that is, how representative the group-level network is for the individual-level networks. We assess this in exactly the same way as in the simulation in Figure 4 by predicting the individual-level networks using the group-level network. Figure 5 shows the ROC curves for each participant and averaged over all participants.

It is clear that the group-level network is a good predictor of the individual-level networks, and there appears to be no indication of a mixture, as in the right panel of Figure 4.

**Figure 6.**
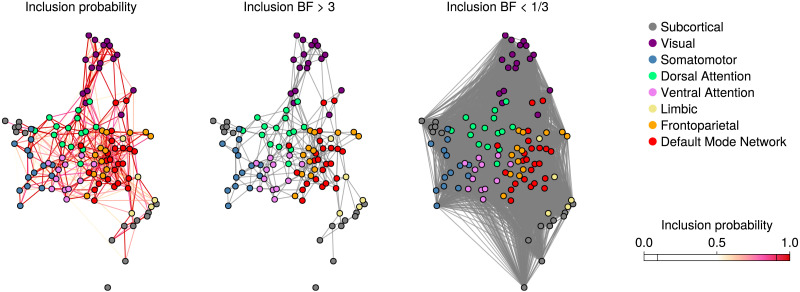
The group network obtained from the Curie-Weiss model. The left panel shows a weighted network where edges represent posterior inclusion probabilities larger than 0.5. The middle and right panels show unweighted networks, where the edges are thresholded by an inclusion Bayes factor of larger than 3 and less than 1/3, respectively.

Next, we examine the group-level network obtained from the Curie-Weiss model. The left panel of Figure 6 shows a weighted network of edge inclusion probabilities. Edges below 0.5 are omitted, and the color indicates the magnitude of the inclusion probability. The middle panel of Figure 6 shows the group-level network where edges are thresholded if their inclusion Bayes factor is less than 3. The inclusion Bayes factor quantifies the change from prior to posterior inclusion probability (Sekulovski et al., 2024). An inclusion Bayes factor larger than 1 indicates evidence for inclusion, less than 1 indicates evidence for exclusion, and exactly 1 is undecided. In practice, broader thresholds are used so that larger than 3 is interpreted as evidence for inclusion, less than 1/3 is seen as evidence for exclusion, and otherwise as insufficient evidence. The right panel of Figure 6 shows for which edges the inclusion Bayes factor was smaller than 1/3. This panel shows that most edges are absent and that the resulting network is quite sparse.

**Figure 7.**
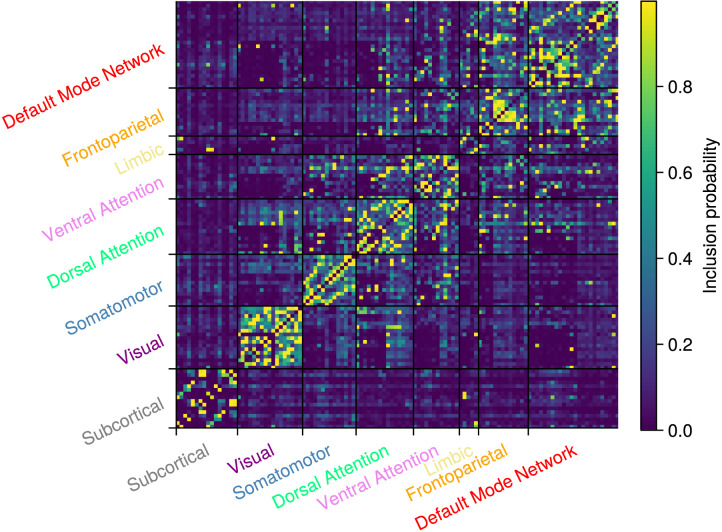
Group-level edge inclusion probabilities.

In both panels, the layout of the nodes is independently generated by the Fruchterman-Reingold algorithm (Fruchterman & Reingold, 1991), which, to some extent, recovers the subdivision into known resting-state networks. Figure 7 shows a heatmap of the edge inclusion probabilities at the group level. The most evidence is for connections within the resting-state networks or modules and comparatively little evidence for connections between them. In addition, the subcortical network and the limbic network appear to be the least connected at the group level.

### Quantifying Individual-Level Heterogeneity

While the previous section explored commonalities among participants by examining group-level outcomes, this section explores individual differences. Our primary focus here is to capture the differences between the individual-level networks and the group-level networks. We examine the heterogeneity at the edge level and aggregated across subnetworks.

**Figure 8.**
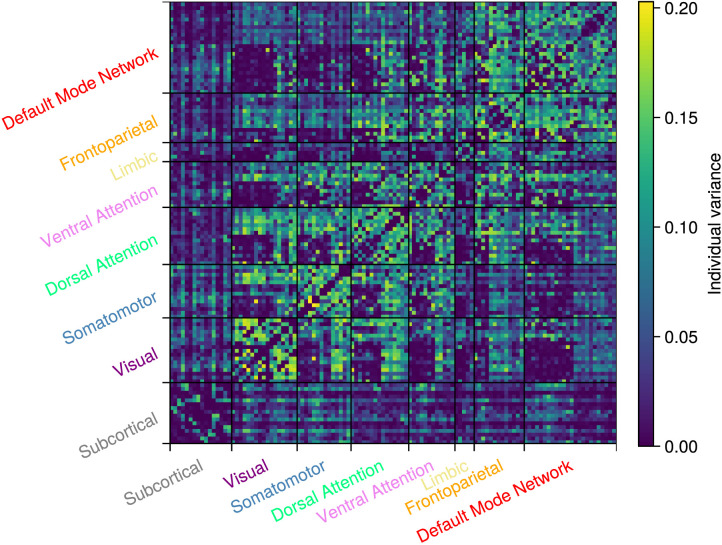
Variance around the group-level edge inclusion probabilities.

We quantify the individual differences by computing, for each edge individually, the variance in the individual-level edge inclusion probabilities using the group-level probability instead of the sample mean. Figure 8 shows the individual differences for all edges.

Contrasting Figures 7 and 8 leads to a variety of results. For example, if we examine the default mode network in Figure 7, it appears that there are two subclusters with a few connections between them. However, Figure 8 shows that the connections between these subclusters vary greatly between participants. This aligns with prior research that found that the Default Mode Network (DMN) consists of multiple subnetworks that vary greatly among people (Braga & Buckner, 2017). In contrast, in the subcortical network in Figure 7, there is no clear indication of subclusters and there are also no large individual differences in Figure 8. There are multiple explanations for this finding. For example, it is known that the reduction in BOLD sensitivity at higher spatial resolution is particularly pronounced in subcortical areas (Lutti et al., 2012; Triantafyllou et al., 2005), which are inherently associated with low BOLD sensitivity due to their increased iron content and marked distance from the receive elements of the head coil (de Hollander, Keuken, van der Zwaag, Forstmann, & Trampel, 2017). Furthermore, subcortical regions are more susceptible to physiological noise compared with cortical areas (Hutton et al., 2011; Kasper et al., 2017; Viviani, 2016). In the group-level analysis, this marked reduction augments the interindividual variability that results in poor differentiation between somatotopy areas (Scholz et al., 2000). Overall, many of the edges for which there is ambiguous evidence at the group level show a high individual variability.

**Figure 9.**
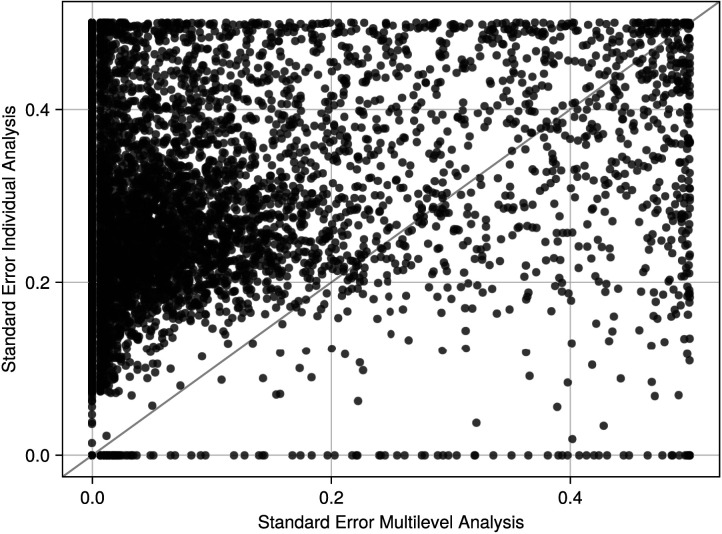
Standard errors in the edge inclusion probabilities of the first participant for the multilevel analysis (*x*-axis) and when the participant is analyzed in isolation (*y*-axis). Most of the standard errors are smaller for the multilevel analysis, which is representative of the information gained from the multilevel approach. Note that the largest possible standard error is 0.5 as the variables are Bernoulli distributed.

Finally, we illustrate a key difference between the multilevel approach and analyzing participants individually. While the results of the multilevel model and individual analyses largely agree, a key difference is that the results of the multilevel model have smaller standard errors compared with the individual analysis because they have access to more information. To illustrate this, we analyzed one participant in isolation and compared the standard errors of the edge inclusion probabilities with the standard errors obtained from the multilevel analysis. Figure 9 visualizes these standard errors from both analyses and shows that the standard errors for the multilevel model are smaller than those obtained from the individual analysis.

## DISCUSSION

Here, we introduced a Bayesian multilevel graphical model to analyze fMRI data. The multilevel model consists of an individual level and a group level, which are jointly estimated. While we made specific choices for both levels, a GGM for the individual level and a Curie-Weiss model for the group level, the key idea behind the multilevel approach is transferable to any individual or group-level model, as long as the group-level model explains all commonalities between the individual-level networks. In addition, we focused on model-agnostic measures (e.g., ROC curves) so that the ideas proposed here can be easily transferred to other individual- or group-level models, as long as they provide edge inclusion probabilities for both the individual and group levels. In two simulations, we showed that the multilevel model outperforms analyzing participants individually or in aggregate in terms of edge recovery and that the multilevel model can identify situations where the participants do not conform to a single group-level model. In a real data analysis, we showed how the multilevel model can be used to study both the group-level and individual differences.

The key reason for using a multilevel model is that it separates within-participant and between-participant variances, thus providing a means to detect Simpson’s paradox. In addition, the multilevel approach has an increased power compared with analyzing participants individually. Another advantage of the multilevel approach is that it can increase the flexibility of the model. For example, it is not a problem if participants’ scans are of different lengths. The individual-level network will adequately account for the difference in information between participants, and this will propagate to the group-level network. In contrast, if participants are analyzed independently, differences in scan length would be reflected in differences in the standard errors, but it would not be straightforward to account for the varying standard errors when subsequently aggregating the results. For example, if one participant’s scan consists of 100 time points and another of 120 time points, then any aggregation method should account for the possibly greater reliability of the longer scan.

A valid concern with estimating a group-level network for resting-state fMRI data is that the group-level network is only interpretable if the parcellations have the same meaning across participants (e.g., see Smith, 2012). To address this, we computed ROC curves to assess how well the individual-level networks were captured by the group-level network (e.g., see Figure 5). While this did not reveal any cause for concern, we feel compelled to point out that an overly narrow focus on the group-level structure has limitations. For example, we found that the group-level network was much sparser than the sparsest individual-level network, and thus, it is plausible that by emphasizing the shared structure, one overlooks important individual differences (see also Mueller et al., 2013; Seitzman et al., 2019). The interpretability of the group-level network also depends on the extent to which participants belong to the same population, and not to different subpopulations. In scenarios where the group-level network is not a good reflection of the sample at hand, such as when clearly distinct subpopulations exist, we advise against using the proposed multilevel approach and recommend extending the model to ensure that the group-level reflects subgroup variations.

We used the GGM for the individual-level networks because it is well understood and widely used. However, a limitation of the GGM is that it ignores the temporal structure in the participant-level data, that is, it treats different time points within the scan of a participant as exchangeable. This clearly need not be the case and may limit the power of our approach (see, e.g., the model used by Fiecas, Cribben, Bahktiari, & Cummine, 2017). One option is to extend the model to include temporal dependencies (see, e.g., Huijsdens, Leeftink, Geerligs, & Hinne, 2024; Kundu & Risk, 2021).

A challenge for graphical modeling is its computational complexity. In our experience, the most computationally demanding aspect has been the estimation of the individual-level networks (as was observed previously, see, e.g., Peterson & Stingo, 2021; Peterson et al., 2015) and not so much the estimation of the group-level network. As such, the multilevel approach is not necessarily more time-consuming than analyzing participants individually.

There is ample opportunity to build on top of the multilevel approach. For example, while we have only looked at the common structure of the individual networks, it is also possible to estimate a weighted group-level network (e.g., see Peterson & Stingo, 2021). In addition, it is possible to specify multiple group networks or to cluster participants into the group networks. Moreover, multilevel models introduce the possibility of using covariates (e.g., age) at the group level. Conceptually, this can be achieved by replacing the thresholds *μ* with a regression equation (see, e.g., Cheng, Levina, Wang, & Zhu, 2014, for a similar approach with the Ising model). Suppose we aim to compare two groups, patients and controls, and denote the (binary) group indicator variable *y*_*n*_, then, we have *μ_en_* = *β*_*e*0_ +*β*_*e*1_*y*_*n*_, where *β*_*e*0_ models the baseline inclusion rate of each edge and *β*_*e*1_ captures the group effect for each edge. This approach can easily be extended to include other covariates, such as age. Many of these ideas are already being applied without a multilevel approach, but this opens the door to all the pitfalls mentioned before. At the same time, there is no technical reason why they cannot be used in conjunction with a multilevel approach.

In summary, it is critical to account for the nested structure in graphical models when observations are nested within participants, as in fMRI research. We believe that multilevel models are key to describing the nested structure and have shown that they can increase statistical power and facilitate the simultaneous exploration of individual differences and shared structure.

## Acknowledgments

Don van den Bergh was supported by a project grant from Amsterdam Brain and Cognition.

## Supporting Information

Supporting information for this article is available at https://doi.org/10.1162/netn_a_00457.

## Author Contributions

Don van den Bergh: Conceptualization; Formal analysis; Methodology; Software; Visualization; Writing – original draft. Linda Douw: Conceptualization; Data curation; Validation; Writing – review & editing. Zarah van der Pal: Conceptualization; Validation; Writing – review & editing. Tessa F. Blanken: Conceptualization; Funding acquisition; Project administration; Validation; Writing – review & editing. Anouk Schrantee: Conceptualization; Funding acquisition; Validation; Writing – review & editing. Maarten Marsman: Conceptualization; Formal analysis; Methodology; Supervision; Writing – review & editing.

## Supplementary Material



## TECHNICAL TERMS

Individual level: The analysis or interpretation that focuses on a single participant or differences between participants.
Group level: The analysis or interpretation that focuses on all participants as a whole, emphasizing what the participants have in common.
Simpson’s paradox: A paradox in statistical analyses where a relation at the individual level is of opposite direction compared with the group level.
Multilevel model: A model that simultaneously describes multiple levels, for example, both the individual level and the group level.
Gaussian graphical model: A model that describes the conditional independence relationships of a set of variables that are assumed to follow a multivariate Gaussian distribution.
Curie-Weiss model: A mean-field approximation to the Ising model, that is, a multivariate distribution over binary variables characterized by main effects and a constant interaction term.
Gibbs sampler: A Markov chain Monte Carlo algorithm that samples from the posterior distribution of the model parameters.
Area under the curve (AUC): A performance metric that quantifies the ability of an algorithm to distinguish between present and absent edges by measuring the area under the receiver operating characteristic (ROC) curve, with higher values indicating better edge detection performance.

## References

[bib2] Barbieri, M. M., & Berger, J. O. (2004). Optimal predictive model selection. Annals of Statistics, 32(3), 870–897. 10.1214/009053604000000238

[bib3] Bassett, D. S., & Sporns, O. (2017). Network neuroscience. Nature Neuroscience, 20(3), 353–364. 10.1038/nn.4502, 28230844 PMC5485642

[bib4] Bezanson, J., Edelman, A., Karpinski, S., & Shah, V. B. (2017). Julia: A fresh approach to numerical computing. SIAM Review, 59(1), 65–98. 10.1137/141000671

[bib5] Braga, R. M., & Buckner, R. L. (2017). Parallel interdigitated distributed networks within the individual estimated by intrinsic functional connectivity. Neuron, 95(2), 457–471. 10.1016/j.neuron.2017.06.038, 28728026 PMC5519493

[bib6] Buckner, R. L., & DiNicola, L. M. (2019). The brain’s default network: Updated anatomy, physiology and evolving insights. Nature Reviews Neuroscience, 20(10), 593–608. 10.1038/s41583-019-0212-7, 31492945

[bib8] Cheng, J., Levina, E., Wang, P., & Zhu, J. (2014). A sparse Ising model with covariates. Biometrics, 70(4), 943–953. 10.1111/biom.12202, 25099186 PMC4425428

[bib9] Chiquet, J., Grandvalet, Y., & Ambroise, C. (2011). Inferring multiple graphical structures. Statistics and Computing, 21, 537–553. 10.1007/s11222-010-9191-2

[bib10] Cho, J. W., Korchmaros, A., Vogelstein, J. T., Milham, M. P., & Xu, T. (2021). Impact of concatenating fMRI data on reliability for functional connectomics. NeuroImage, 226, 117549. 10.1016/j.neuroimage.2020.117549, 33248255 PMC7983579

[bib12] de Hollander, G., Keuken, M. C., van der Zwaag, W., Forstmann, B. U., & Trampel, R. (2017). Comparing functional MRI protocols for small, iron-rich basal ganglia nuclei such as the subthalamic nucleus at 7 T and 3 T. Human Brain Mapping, 38(6), 3226–3248. 10.1002/hbm.23586, 28345164 PMC6867009

[bib13] Dempster, A. P. (1972). Covariance selection. Biometrics, 157–175. 10.2307/2528966

[bib14] Dichter, G. S., Gibbs, D., & Smoski, M. J. (2015). A systematic review of relations between resting-state functional-MRI and treatment response in major depressive disorder. Journal of Affective Disorders, 172, 8–17. 10.1016/j.jad.2014.09.028, 25451389 PMC4375066

[bib16] Fawcett, T. (2006). An introduction to ROC analysis. Pattern Recognition Letters, 27(8), 861–874. 10.1016/j.patrec.2005.10.010

[bib17] Fiecas, M., Cribben, I., Bahktiari, R., & Cummine, J. (2017). A variance components model for statistical inference on functional connectivity networks. NeuroImage, 149, 256–266. 10.1016/j.neuroimage.2017.01.051, 28130192

[bib19] Fruchterman, T. M. J., & Reingold, E. M. (1991). Graph drawing by force-directed placement. Software: *Practice and Experience*, 21(11), 1129–1164. 10.1002/spe.4380211102

[bib20] Gordon, E. M., Laumann, T. O., Adeyemo, B., & Petersen, S. E. (2017). Individual variability of the system-level organization of the human brain. Cerebral Cortex, 27(1), 386–399. 10.1093/cercor/bhv239, 26464473 PMC5939195

[bib21] Gratton, C., Laumann, T. O., Nielsen, A. N., Greene, D. J., Gordon, E. M., Gilmore, A. W., … Petersen, S. E. (2018). Functional brain networks are dominated by stable group and individual factors, not cognitive or daily variation. Neuron, 98(2), 439–452. 10.1016/j.neuron.2018.03.035, 29673485 PMC5912345

[bib22] Harms, M. P., Somerville, L. H., Ances, B. M., Andersson, J., Barch, D. M., Bastiani, M., … Yacoub, E. (2018). Extending the Human Connectome Project across ages: Imaging protocols for the lifespan development and aging projects. NeuroImage, 183, 972–984. 10.1016/j.neuroimage.2018.09.060, 30261308 PMC6484842

[bib23] Huijsdens, H., Leeftink, D., Geerligs, L., & Hinne, M. (2024). Robust inference of dynamic covariance using Wishart processes and sequential Monte Carlo. Entropy, 26(8), 695. 10.3390/e26080695, 39202165 PMC11353982

[bib24] Hutton, C., Josephs, O., Stadler, J., Featherstone, E., Reid, A., Speck, O., … Weiskopf, N. (2011). The impact of physiological noise correction on fMRI at 7 T. NeuroImage, 57(1), 101–112. 10.1016/j.neuroimage.2011.04.018, 21515386 PMC3115139

[bib25] Kac, M. (1968). Mathematical mechanisms of phase transition. In M. Chrétilin, E. P. Gross, & S. Deser (Eds.), *Statistical mechanics of phase transitions and superfluidity*. New York: Gordon and Breach Science Publisher.

[bib26] Kasper, L., Bollmann, S., Diaconescu, A. O., Hutton, C., Heinzle, J., Iglesias, S., … Stephan, K. E. (2017). The PhysIO toolbox for modeling physiological noise in fMRI data. Journal of Neuroscience Methods, 276, 56–72. 10.1016/j.jneumeth.2016.10.019, 27832957

[bib27] Kass, R. E., & Raftery, A. E. (1995). Bayes factors. Journal of the American Statistical Association, 90(430), 773–795. 10.1080/01621459.1995.10476572

[bib28] Kievit, R. A., Frankenhuis, W. E., Waldorp, L. J., & Borsboom, D. (2013). Simpson’s paradox in psychological science: A practical guide. Frontiers in Psychology, 4, 513. 10.3389/fpsyg.2013.00513, 23964259 PMC3740239

[bib29] Krull, J. L., & MacKinnon, D. P. (2001). Multilevel modeling of individual and group level mediated effects. Multivariate Behavioral Research, 36(2), 249–277. 10.1207/S15327906MBR3602_06, 26822111

[bib30] Kundu, S., & Risk, B. B. (2021). Scalable Bayesian matrix normal graphical models for brain functional networks. Biometrics, 77(2), 439–450. 10.1111/biom.13319, 32569385

[bib31] Lauritzen, S. L. (1996). *Graphical models* (Vol. 17). Clarendon Press. 10.1093/oso/9780198522195.001.0001

[bib32] Lenkoski, A. (2013). A direct sampler for G-Wishart variates. Stat, 2(1), 119–128. 10.1002/sta4.23

[bib35] Lutti, A., Stadler, J., Josephs, O., Windischberger, C., Speck, O., Bernarding, J., … Weiskopf, N. (2012). Robust and fast whole brain mapping of the RF transmit field B1 at 7T. PLoS One, 7(3), e32379. 10.1371/journal.pone.0032379, 22427831 PMC3299646

[bib36] Mangalam, M., & Kelty-Stephen, D. G. (2021). Point estimates, Simpson’s paradox, and nonergodicity in biological sciences. Neuroscience & Biobehavioral Reviews, 125, 98–107. 10.1016/j.neubiorev.2021.02.017, 33621638

[bib38] Marsman, M., Tanis, C., Bechger, T., & Waldorp, L. (2019). Network psychometrics in educational practice: Maximum likelihood estimation of the Curie-Weiss model. Theoretical and Practical Advances in Computer-Based Educational Measurement, 93–120. 10.1007/978-3-030-18480-3_5

[bib39] Mueller, S., Wang, D., Fox, M. D., Yeo, B. T. T., Sepulcre, J., Sabuncu, M. R., … Liu, H. (2013). Individual variability in functional connectivity architecture of the human brain. Neuron, 77(3), 586–595. 10.1016/j.neuron.2012.12.028, 23395382 PMC3746075

[bib40] Peterson, C. B., & Stingo, F. C. (2021). Bayesian estimation of single and multiple graphs. In *Handbook of bayesian variable selection* (pp. 327–348). Chapman and Hall/CRC. 10.1201/9781003089018-14

[bib41] Peterson, C. B., Stingo, F. C., & Vannucci, M. (2015). Bayesian inference of multiple gaussian graphical models. Journal of the American Statistical Association, 110(509), 159–174. 10.1080/01621459.2014.896806, 26078481 PMC4465207

[bib42] Pollet, T. V., Stulp, G., Henzi, S. P., & Barrett, L. (2015). Taking the aggravation out of data aggregation: A conceptual guide to dealing with statistical issues related to the pooling of individual-level observational data. American Journal of Primatology, 77(7), 727–740. 10.1002/ajp.22405, 25810242

[bib43] Roberts, R. P., Hach, S., Tippett, L. J., & Addis, D. R. (2016). The Simpson’s paradox and fMRI: Similarities and differences between functional connectivity measures derived from within-subject and across-subject correlations. NeuroImage, 135, 1–15. 10.1016/j.neuroimage.2016.04.028, 27101735

[bib44] Robinson, W. S. (2009). Ecological correlations and the behavior of individuals. International Journal of Epidemiology, 38(2), 337–341. 10.1093/ije/dyn357, 19179346

[bib45] Roverato, A. (2002). Hyper inverse Wishart distribution for non-decomposable graphs and its application to Bayesian inference for Gaussian graphical models. Scandinavian Journal of Statistics, 29(3), 391–411. 10.1111/1467-9469.00297

[bib46] Schad, D. J., Nicenboim, B., & Vasishth, S. (2024). Data aggregation can lead to biased inferences in Bayesian linear mixed models and Bayesian analysis of variance. Pyschological Methods. 10.1037/met0000621, 38271007

[bib47] Schaefer, A., Kong, R., Gordon, E. M., Laumann, T. O., Zuo, X.-N., Holmes, A. J., … Yeo, B. T. T. (2018). Local-global parcellation of the human cerebral cortex from intrinsic functional connectivity mri. Cerebral Cortex, 28(9), 3095–3114. 10.1093/cercor/bhx179, 28981612 PMC6095216

[bib48] Scholz, V. H., Flaherty, A. W., Kraft, E., Keltner, J. R., Kwong, K. K., Chen, Y. I., … Jenkins, B. G. (2000). Laterality, somatotopy and reproducibility of the basal ganglia and motor cortex during motor tasks. Brain Research, 879(1–2), 204–215. 10.1016/S0006-8993(00)02749-9, 11011024

[bib50] Seitzman, B. A., Gratton, C., Laumann, T. O., Gordon, E. M., Adeyemo, B., Dworetsky, A., … Petersen, S. E. (2019). Trait-like variants in human functional brain networks. Proceedings of the National Academy of Sciences, 116(45), 22851–22861. 10.1073/pnas.1902932116, 31611415 PMC6842602

[bib51] Sekulovski, N., Keetelaar, S., Huth, K., Wagenmakers, E.-J., van Bork, R., van den Bergh, D., & Marsman, M. (2024). Testing conditional independence in psychometric networks: An analysis of three Bayesian methods. Multivariate Behavioral Research, 59(5), 913–933. 10.1080/00273171.2024.2345915, 38733319

[bib52] Simpson, E. H. (1951). The interpretation of interaction in contingency tables. Journal of the Royal Statistical Society: Series B (Methodological), 13(2), 238–241. 10.1111/j.2517-6161.1951.tb00088.x

[bib53] Smith, S. M. (2012). The future of FMRI connectivity. NeuroImage, 62(2), 1257–1266. 10.1016/j.neuroimage.2012.01.022, 22248579

[bib54] Tian, Y., Margulies, D. S., Breakspear, M., & Zalesky, A. (2020). Topographic organization of the human subcortex unveiled with functional connectivity gradients. Nature Neuroscience, 23(11), 1421–1432. 10.1038/s41593-020-00711-6, 32989295

[bib55] Tijhuis, F. B., Schepers, M., Centeno, E., Maciel, B., Douw, L., & Nobrega Santos, F. (2022). *Human Connectome Project resting-state fMRI Connectivity Matrices (Young Adult + Aging)*. Zenodo. 10.5281/zenodo.6770120

[bib56] Tijhuis, F. B., Schepers, M., Centeno, E., Maciel, B., Douw, L., & Nobrega Santos, F. (2023). *Human Connectome Project rfMRI repository*. GitHub repository Retrieved from https://github.com/floristijhuis/HCP-rfMRI-repository (Accessed 2023-6-2).

[bib57] Triantafyllou, C., Hoge, R. D., Krueger, G., Wiggins, C. J., Potthast, A., Wiggins, G. C., & Wald, L. L. (2005). Comparison of physiological noise at 1.5 T, 3 T and 7 T and optimization of fMRI acquisition parameters. NeuroImage, 26(1), 243–250. 10.1016/j.neuroimage.2005.01.007, 15862224

[bib58] Tsai, K., Koyejo, O., & Kolar, M. (2022). Joint Gaussian graphical model estimation: A survey. Wiley Interdisciplinary Reviews: Computational Statistics, 14(6), e1582. 10.1002/wics.1582

[bib59] Uhler, C. (2018). Gaussian graphical models. In *Handbook of graphical models* (pp. 217–238). CRC Press. 10.1201/9780429463976-9

[bib60] van der Pal, Z., Douw, L., Genis, A., van den Bergh, D., Marsman, M., Schrantee, A., & Blanken, T. (2024). Tell me why? A scoping review on the fundamental building blocks of fMRI networks. PsyArxiv Preprint. 10.1016/j.nicl.2025.103785 PMC12264222 40245454

[bib61] van Doorn, J., van den Bergh, D., Böhm, U., Dablander, F., Derks, K., Draws, T., … Wagenmakers, E.-J. (2021). The JASP guidelines for conducting and reporting a Bayesian analysis. Psychonomic Bulletin & Review, 28, 813–826. 10.3758/s13423-020-01798-5, 33037582 PMC8219590

[bib62] Vehtari, A., Gelman, A., Simpson, D., Carpenter, B., & Bürkner, P.-C. (2021). Rank-normalization, folding, and localization: An improved *R* for assessing convergence of MCMC (with discussion). Bayesian Analysis, 16(2), 667–718. 10.1214/20-BA1221

[bib63] Viviani, R. (2016). A digital atlas of middle to large brain vessels and their relation to cortical and subcortical structures. Frontiers in Neuroanatomy, 10, 12. 10.3389/fnana.2016.00012 26924965 PMC4756124

[bib64] Vogels, L., Mohammadi, R., Schoonhoven, M., & Birbil, Ş. İ. (2024). Bayesian structure learning in undirected Gaussian graphical models: Literature review with empirical comparison. Journal of the American Statistical Association, 119(548), 3164–3182. 10.1080/01621459.2024.2395504

[bib65] Wang, H. (2012). Bayesian graphical lasso models and efficient posterior computation. Bayesian Analysis, 7(4), 867–886. 10.1214/12-BA729

[bib66] Wang, H. (2015). Scaling it up: Stochastic search structure learning in graphical models. *Bayesian* Analysis, 10(2), 351–377. 10.1214/14-BA916

[bib67] Watkins, I. J., & Martire, K. A. (2015). Generalized linear mixed models for deception research: Avoiding problematic data aggregation. Psychology, Crime & Law, 21(9), 821–835. 10.1080/1068316X.2015.1054384

[bib68] Xiao, Y., Wen, T. H., Kupis, L., Eyler, L. T., Taluja, V., Troxel, J., … Courchesne, E. (2023). Atypical functional connectivity of temporal cortex with precuneus and visual regions may be an early-age signature of ASD. Molecular Autism, 14(1), 11. 10.1186/s13229-023-00543-8, 36899425 PMC10007788

[bib69] Yeo, B. T. T., Krienen, F. M., Sepulcre, J., Sabuncu, M. R., Lashkari, D., Hollinshead, M., … Buckner, R. L. (2011). The organization of the human cerebral cortex estimated by intrinsic functional connectivity. Journal of Neurophysiology, 106(3), 1125–1165. 10.1152/jn.00338.2011, 21653723 PMC3174820

[bib70] Zimmermann, M. L. M., Breedt, L. C., Centeno, E. G. Z., Reijneveld, J. C., Santos, F. A. N., Stam, C. J., … Douw, L. (2024). The relationship between pathological brain activity and functional network connectivity in glioma patients. Journal of Neuro-Oncology, 166(3), 523–533. 10.1007/s11060-024-04577-7, 38308803 PMC10876827

