# Supplementary material for: Jointly estimating individual and group networks from fMRI data
Source: Netw Neurosci. 2025 Jul 29;9(3):896–912. doi: 10.1162/netn_a_00457 (PMC12543299; doi:10.1162/netn_a_00457)
Supplement: Supplementary file 1 [file netn-9-3-896-s001.pdf]

Supplementary Materials for: Jointly Estimating Individual and Group Networks from fMRI Data

A Additional Simulation Results

Figures A.1 and A.2 show the simulation results including the condition omitted in the main text.

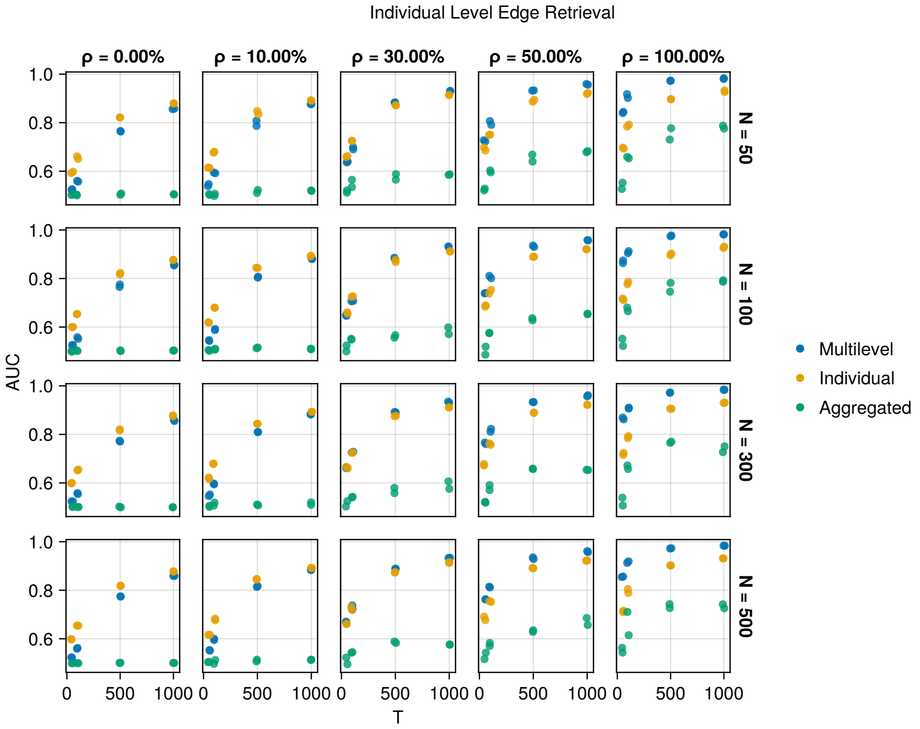

Figure A.1: AUC for edge retrieval of the individual-level networks (y-axis) against the number of time points (x-axis) for the three methods on simulated data (colors and shapes). The top row is identical to the results shown in the main text, the other rows show the remaining simulation conditions.

B The Gaussian Graphical Model

B.1 Prior work on group-level networks

This section reviews some earlier work on Gaussian graphical models for nested data, (for more thorough reviews, see Peterson & Stingo, 2021; Tsai, Koyejo, & Kolar, 2022).

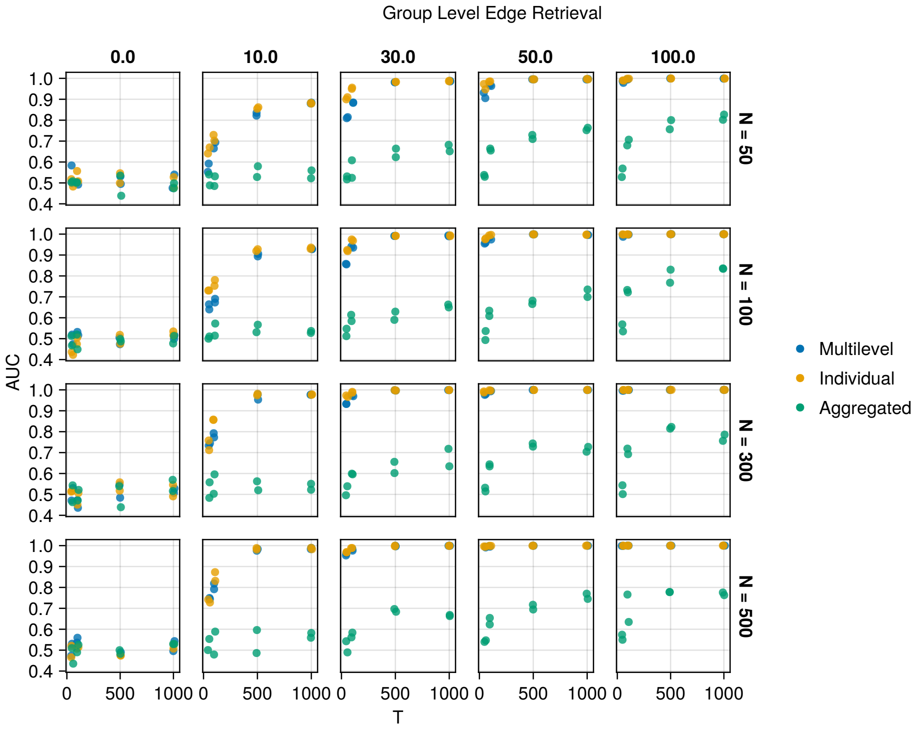

Figure A.2: AUC for edge retrieval of the group-level networks (y-axis) against the number of time points (x-axis) for the three methods on simulated data (colors and shapes). The top row is identical to the results shown in the main text, the other rows show the remaining simulation conditions.

A popular approach is the graphical horseshoe (Busatto & Stingo, 2023; Li, Craig, & Bhadra, 2019; Lingjærde, Fairfax, Richardson, & Ruffieux, 2022), which uses local and global shrinkage parameters for each individual. These individual-level shrinkage parameters are related by their prior distributions, allowing information to be shared across individuals. While the graphical horseshoe successfully pools data from different individuals, it makes two major concessions along the way. First, there is no explicit model for the group-level network. Thus, although information is pooled across individuals, this does not help to generalize over participants. Second, while the graphical horseshoe shrinks parameters to zero, they are never exactly zero, and thus all the estimated individual networks are fully connected (i.e., all edges are present). While thresholding small parameter estimates may seem like an obvious solution, this foregoes any uncertainty estimation (e.g., what is the probability that an edge is present or absent?). This is problematic because the zeros determine the structure of the network and are key to building theory and informing practical decisions.

An alternative and straightforward approach is to use a Beta-binomial prior (Colclough et al., 2018). This Beta-binomial is often used as a model prior in linear regression and has desirable properties for multiple testing (e.g., controlling the false-positive rate; Scott & Berger, 2006).

B.2 The prior on γ

The natural device for constructing a group network is the prior distribution on the edge inclusion parameters. This approach was taken before to compare multiple groups (Peterson, Stingo, & Vannucci, 2015). Whereas Peterson et al. (2015) used the edge inclusion prior to contrast multiple groups (consisting of multiple participants), we use it to model individual participants directly. Here we summarize this approach and introduce our Curie-Weiss distribution as an alternative. To simplify the notation and comparison with Peterson et al. (2015), we introduce some additional notation. Let $E = {}^{P(P-1)}/_{2}$ denote the number of edges. We use $\mathbf{\Gamma}$ to denote the $E \times N$ matrix that is obtained by concatenating the vectorization of the lower triangle of the graph of each individual and denote an individual edge with e. For example, the edge between nodes i and j for participant n is denoted γ_{ijn} in the section discussing the Gaussian graphical model and Γ_{en} from here on.

Peterson et al. (2015) use a Markov Random Field (MRF) distribution to model the group structure. The MRF distribution consists of two parameters; a vector that models the edge inclusions, $\boldsymbol{\mu}$, and a matrix that captures the similarity between groups (or individuals), $\mathbf{\Theta}$. Their MRF distribution is given by

$$p\left(\boldsymbol{\gamma}_e \mid \mu_e, \mathbf{\Theta}\right) = Z(\mu_e, \mathbf{\Theta})^{-1} \exp\left(\mu_e \mathbf{1}^T \boldsymbol{\gamma}_e + \boldsymbol{\gamma}_e^T \mathbf{\Theta} \boldsymbol{\gamma}_e\right),$$

where $\boldsymbol{\gamma}_e$ indicates the vector of edge inclusions for all participants for a particular edge e (i.e., row e of $\mathbf{\Gamma}$). These vectors of edge inclusions are assumed to be independent a-priori and thus the joint distribution is given by the product:

$$p\left(\mathbf{\Gamma} \mid \boldsymbol{\mu}, \mathbf{\Theta}\right) = \prod_{e=1}^{E} p\left(\boldsymbol{\gamma}_e \mid \mu_e, \mathbf{\Theta}\right).$$

As is common for MRF distributions, the normalizing constant is the sum of all possible states:

$$Z(\mu_e, \mathbf{\Theta}) = \sum_{\boldsymbol{\gamma}_e \in \{0,1\}^K} \exp\left(\mu_e \mathbf{1}^T \boldsymbol{\gamma}_e + \boldsymbol{\gamma}_e^T \mathbf{\Theta} \boldsymbol{\gamma}_e\right).$$

The number of terms in the normalizing constant grows exponentially in the number of participants and becomes practically infeasible to compute for more than 15 participants.

To alleviate the computational complexity of the MRF distribution by Peterson et al. (2015), we propose a Curie-Weiss distribution as a prior instead. The Curie-Weiss distribution is given by

$$p\left(\boldsymbol{\gamma}_n \mid \boldsymbol{\mu}, \sigma\right) = Z(\mu, \sigma)^{-1} \exp\left(\boldsymbol{\mu}^T \boldsymbol{\gamma}_n + \frac{\sigma}{E}\left(\boldsymbol{\gamma}_n^T \boldsymbol{\gamma}_n\right)^2\right),$$

where $\boldsymbol{\gamma}_n$ is the vector of all edge inclusions for a single participant (i.e., column n of $\boldsymbol{\Gamma}$). Note that $\boldsymbol{\gamma}_n^T \boldsymbol{\gamma}_n$ is equal to $(\gamma_n^+)^2$. The joint distribution is given by the product over all participants and can be expressed as

$$p\left(\boldsymbol{\Gamma} \mid \boldsymbol{\mu}, \sigma\right) = Z(\boldsymbol{\mu}, \sigma)^{-N} \exp\left(\boldsymbol{\mu}^T \boldsymbol{\Gamma} \mathbf{1} + \frac{\sigma}{E} \mathbf{1}^T \boldsymbol{\Gamma}^T \boldsymbol{\Gamma} \mathbf{1}\right).$$

While the normalizing constant of the Curie-Weiss prior is also defined as the reciprocal of the sum over all possible states, this sum has a closed-form solution in terms of elementary symmetric functions. In particular, this implies that rather than enumerating all possible states of the network, we only need to enumerate all possible degrees (i.e., 0, 1, ..., E). For example, suppose we have a network with 3 nodes and order the terms in the normalizing constant by the degree of the enumerated network. Then we obtain

$$\begin{aligned}
Z(\boldsymbol{\mu}, \sigma) = 1 + & & & \text{(empty network)}\\
& (\exp(\mu_1) + \exp(\mu_2) + \exp(\mu_3)) & \exp\left(\frac{\sigma}{E} 1^2\right) + & \text{(degree 1)}\\
& (\exp(\mu_1 + \mu_2) + \exp(\mu_1 + \mu_3) + \exp(\mu_2 + \mu_3)) & \exp\left(\frac{\sigma}{E} 2^2\right) + & \text{(degree 2)}\\
& \exp(\mu_1 + \mu_2 + \mu_3) & \exp\left(\frac{\sigma}{E} 3^2\right) & \text{(degree 3)}.
\end{aligned}$$

Each term on the left is given by an elementary function of appropriate order, for example, $e_3\left(\exp(\boldsymbol{\mu})\right)$ would give the fine line. For more details on the elementary symmetric functions, see Chapters 2 and 3 of Egge (2019). For an arbitrary size network, the normalizing constant of the Curie-Weiss model simplifies to

$$Z(\boldsymbol{\mu}, \sigma) = \sum_{s=0}^{E} e_s\left(\exp(\boldsymbol{\mu})\right) \exp\left(\frac{\sigma}{E} s^2\right),$$

where $\exp(\boldsymbol{\mu})$ is taken element-wise and $e_s(\boldsymbol{\mu})$ denotes the elementary symmetric function of order s of the vector $\boldsymbol{\mu}$. This alleviates the computational complexity from growing exponentially in the number of edges to growing quadratically in the number of edges.

The Curie-Weiss distribution and the MRF distribution have in common that the parameter μ strongly relates to the inclusion probability of a particular edge. In fact, when σ and $\boldsymbol{\Theta}$ tend to zero both priors reduce to a Bernoulli model with $p(\gamma_e = 1) = 1/(1 + \exp(-\mu_e))$. However, there are some important substantial differences between the Curie-Weiss prior and the MRF distribution. The MRF distribution assumes that different rows (i.e., edges) are independent given $\boldsymbol{\mu}$ and $\boldsymbol{\Theta}$. In contrast, the Curie-Weiss prior assumes that different columns (i.e., participants) are independent given the parameters $\boldsymbol{\mu}$ and σ. These assumptions have computational consequences when sampling from the posterior distributions of the individual networks. In particular, in a Gibbs sampler for the Curie-Weiss prior the individual network parameters can be sampled in parallel, as they are independent conditional on μ and σ. For the MRF distribution, it is possible to sample different edges of different individual networks in parallel, although this requires a considerably more sophisticated approach as different threads or processes have to communicate to figure out which edges of which individuals are available to update.

Neither the Curie-Weiss distribution nor the MRF distribution explicitly models a vector of binary parameters that represents the group-level network. Instead, we assume that their marginal expectations approximate the group-level network. Obtaining this expectation is relatively straightforward to do by Monte Carlo simulation, but for the Curie-Weiss prior it is also available analytically:

$$p(\gamma_e = 0 \mid \boldsymbol{\mu}, \sigma) = \sum_{\boldsymbol{\gamma}_{-e} \in \{0,1\}^{E-1}} p(\gamma_e = 0, \boldsymbol{\gamma}_{\boldsymbol{-e}} \mid \boldsymbol{\mu}, \sigma) \tag{1}$$

$$= \frac{\sum_{s=0}^{E-1} e_s(\exp(\boldsymbol{\mu}_{-e})) \exp\left(\frac{\sigma}{E} s^2\right)}{\sum_{s=0}^{E} e_s(\exp(\boldsymbol{\mu})) \exp\left(\frac{\sigma}{E} s^2\right)} \tag{2}$$

Finally, one might wonder why we use the Curie-Weiss model instead of the more general Ising model. The primary reason is that the Ising model adds too many parameters that do not clearly benefit the interpretation of the group-level model. In addition, the Ising model is computationally expensive ($\mathcal{O}(P^4)$). [1]

Combining the individual-level GGMs with the group-level Curie-Weiss distribution, we obtain the following expression for the joint posterior distribution.

[1]The number of edges in the group network is $P(P-1)/2$ and the number of interaction parameters in the Ising model is $E(E-1)/2$, so the number of parameters is of quartic order, not to mention any algorithm to estimate these.

$$p\left(\boldsymbol{\Omega}_1,\ldots,\boldsymbol{\Omega}_K,\boldsymbol{\Gamma}\mid\boldsymbol{X}_1,\ldots,\boldsymbol{X}_K\right)\propto\left[\prod_{k=1}^{K}(2\pi)^{-\frac{N_k}{2}}\det\left(\boldsymbol{\Omega}_k\right)^{\frac{N_k}{2}}\exp\left(-\frac{N}{2}\operatorname{Tr}\left(\boldsymbol{\Omega}_k\boldsymbol{S}_k\right)\right)\right.$$
$$\left.\times\prod_{p=1}^{P}\mathcal{E}\left(\omega_{ppk}\mid\lambda\right)\prod_{p\prime<p}\mathcal{N}\left(\omega_{p\prime pk}\mid 0,\left(v_1^{\gamma_{p\prime pk}}v_0^{1-\gamma_{p\prime pk}}\right)^2\right)\right]$$
$$\times\frac{\exp\left(\boldsymbol{\mu}^T\boldsymbol{\Gamma}\mathbf{1}+\frac{\sigma}{E}\mathbf{1}^T\boldsymbol{\Gamma}^T\boldsymbol{\Gamma}\mathbf{1}\right)}{\sum_{s=0}^{E}e_s\left(\exp(\boldsymbol{\mu})\right)\exp\left(\frac{\sigma}{E}s^2\right)}\mathcal{N}_+\left(\sigma\mid 0,1\right)$$

B.3 Implementation

Here we outline a Gibbs sampler for the joint posterior described before. We first derive the full conditionals for the Gaussian graphical model with spike-and-slab priors, and afterward for the Curie-Weiss distribution.

Using the spike and slab approach we can simulate from the posterior distribution for $\boldsymbol{\Omega}$ with a Gibbs sampler in the following manner. As before, we use $\boldsymbol{S}$ to denote the sample covariance matrix and partition the precision matrix and sample covariance matrix in the following manner.

$$\boldsymbol{\Omega}=\begin{bmatrix}\boldsymbol{\Omega}_{-p,-p} & \boldsymbol{\Omega}_{-p,p}\\ \boldsymbol{\Omega}_{p,-p} & \omega_{p,p}\end{bmatrix},\quad \boldsymbol{S}=\begin{bmatrix}\boldsymbol{S}_{-p,-p} & \boldsymbol{S}_{-p,p}\\ \boldsymbol{S}_{p,-p} & s_{p,p}\end{bmatrix}.$$

Here, we use a negative index to denote all indices except that index, for example, $\boldsymbol{\Omega}_{-p,-p}$ is the submatrix without row and column p. Using the properties of block matrices for determinants and traces, we obtain

$$p\left(\boldsymbol{\Omega}_{p,-p},\omega_{p,p}|\boldsymbol{X},\boldsymbol{\Omega}_{-p,-p}\right)\propto\left(\omega_{p,p}-\boldsymbol{\omega}_{p,-p}^{'}\boldsymbol{\Omega}_{-p,-p}\boldsymbol{\omega}_{p,-p}\right)^{\frac{N}{2}}$$
$$\times\exp\left(-\frac{1}{2}\left(\boldsymbol{\omega}_{p,-p}^{'}\boldsymbol{V}_p^{-1}\boldsymbol{\omega}_{p,-p}+2\boldsymbol{S}_{p,-p}^{'}\boldsymbol{\omega}_{p,-p}+(s22+\lambda)\omega_{p,p}\right)\right)$$

where $\boldsymbol{V}_p=\operatorname{diag}\left(\boldsymbol{v}_{-p}\right)$. Next, we apply a change of variables from $(\boldsymbol{u}=\boldsymbol{\omega}_{p,-p}, v=\omega_{p,p}-\boldsymbol{\omega}_{p,-p}^{'}\boldsymbol{\Omega}_{-p,-p}\boldsymbol{\omega}_{p,-p})$ and recognize the following conditional distributions:

$$p\left(\boldsymbol{u}\mid\boldsymbol{X},\boldsymbol{\Omega}_{-p,-p}\right)\propto\mathcal{N}\left(-\boldsymbol{C}\boldsymbol{s}_{-p,p},\boldsymbol{C}\right)$$
$$p\left(v\mid\boldsymbol{X},\boldsymbol{\Omega}_{-p,-p}\right)\propto\mathcal{G}\left(\frac{N}{2}-1,\frac{s_{p,p}+\lambda}{2}\right),$$

where $\boldsymbol{C}=\left(\left(s_{pp}+\lambda\right)\left(\boldsymbol{\Omega}_{-p,-p}\right)^{-1}+\boldsymbol{V}_p^{-1}\right)^{-1}$ and $\mathcal{G}\left(a,b\right)$ denotes the Gamma distribution with shape a and scale b.

The conditional distribution of the indicator variables is straightforward, as these only appear in the prior distribution of ω_{ij}. We have

$$p\left(\gamma_{ij}=1\mid\boldsymbol{\Omega}\right)=\frac{\mathcal{N}\left(\omega_{ij}\mid 0,v_1^2\right)p\left(\gamma_{ij}\right)}{\mathcal{N}\left(\omega_{ij}\mid 0,v_1^2\right)p\left(\gamma_{ij}\right)+\mathcal{N}\left(\omega_{ij}\mid 0,v_0^2\right)\left(1-p\left(\gamma_{ij}\right)\right)}.$$

The Gibbs sampler for the precision matrix involves three hyperparameters, λ the scale of the exponential prior on the diagonal elements of the precision matrix, and the spike and slab variances v_0 and v_1. Throughout the simulations and analyses, we fixed λ to 0.1 and v_0 and v_1 to .1 and 10 respectively. In Appendix C we explore the impact of the value of v_0.

Next, we discuss the full conditionals for the Curie-Weiss distribution. To sample from the posterior distribution of $\boldsymbol{\mu}$ we use the Gibbs sampler by Maris, Bechger, and Martin (2015), which makes use of the following well-known property of elementary symmetric functions (Baker & Harwell, 1996; Fischer, 1974):

$$e_s\left(\boldsymbol{\mu}\right) = e_{s-1}\left(\boldsymbol{\mu}_{-i}\right)\mu_i + e_s\left(\boldsymbol{\mu}_{-i}\right).$$

Using this property, we can isolate μ_i in the normalizing constant of the Curie-Weiss model

$$\begin{aligned}\sum_{s=0}^{E} e_s\left(\exp(\boldsymbol{\mu})\right)\exp\left(\frac{\sigma}{E}s^2\right) &= \sum_{s=0}^{E}\left(e_{s-1}\left(\exp\left(\boldsymbol{\mu}_{-i}\right)\right) + e_s\left(\exp\left(\boldsymbol{\mu}_{-i}\right)\right)\exp\left(\mu_i\right)\right)\exp\left(\frac{\sigma}{E}s^2\right)\\ &= \sum_{s=0}^{E}\left(e_{s-1}\left(\exp\left(\boldsymbol{\mu}_{-i}\right)\right)\exp\left(\frac{\sigma}{E}s^2\right)\right)\\ &\quad + \exp\left(\mu_i\right)\sum_{s=0}^{E}\left(e_s\left(\exp\left(\boldsymbol{\mu}_{-i}\right)\right)\right)\exp\left(\frac{\sigma}{E}s^2\right)\end{aligned}$$

From here, the conditional distribution for μ_i is proportional to

$$\begin{aligned}p\left(\mu_i \mid \boldsymbol{\Gamma}, \boldsymbol{\mu}_{-i}, \sigma\right) &\propto \frac{\exp\left(\boldsymbol{\mu}^T\boldsymbol{\Gamma}\mathbf{1} + \frac{\sigma}{E}\mathbf{1}^T\boldsymbol{\Gamma}^T\boldsymbol{\Gamma}\mathbf{1}\right)}{\left(\sum_{s=0}^{E} e_s\left(\exp(\boldsymbol{\mu})\right)\exp\left(\frac{\sigma}{E}s^2\right)\right)^N}\; p\left(\mu_i\right)\\ &\propto \exp\left(s_i\mu_i\right)\left(\sum_{s=0}^{E}\left(e_{s-1}\left(\exp\left(\boldsymbol{\mu}_{-i}\right)\right)\exp\left(\frac{\sigma s^2}{E}\right)\right)\right.\\ &\quad \left. + \exp\left(\mu_i\right)\sum_{s=0}^{E}\left(e_s\left(\exp\left(\boldsymbol{\mu}_{-i}\right)\right)\right)\exp\left(\frac{\sigma s^2}{E}\right)\right)^{-N} p\left(\mu_i\right)\\ &\propto \frac{\exp\left(s_i\mu_i\right)}{\left(1 + c_i\exp\mu_i\right)^K}\; p\left(\mu_i\right)\\ &\propto \mathcal{B}'\left(\exp(\mu_i); s_i, K - s_i, 1, 1/c\right)\; p\left(\mu_i\right)\end{aligned}$$

where $s_i = \sum_{k=0}^{K}\Gamma_{ik}$, the total number of participants for which edge i is included, $c_i = \frac{\sum_{s=0}^{E} e_{s-1}(\exp(\boldsymbol{\mu}_{-i}))\exp\left(\frac{\sigma}{E}s^2\right)}{\sum_{s=0}^{E} e_s(\exp(\boldsymbol{\mu}_{-i}))\exp\left(\frac{\sigma}{E}s^2\right)}$, and $\mathcal{B}'\left(x; \alpha, \beta, p, q\right)$ refers to the generalized beta-prime distribution, defined as

$$\mathcal{B}'\left(x; \alpha, \beta, p, q\right) = \frac{p\left(\frac{x}{q}\right)^{\alpha p - 1}\left(1 + \left(\frac{x}{q}\right)^p\right)^{-\alpha-\beta}}{qB(\alpha, \beta)}.$$

To simulate from the beta-prime distribution, we make use of its compound representation $\mathcal{B}'\left(x;\alpha,\beta,1,q\right) = \int_0^\infty \text{Gamma}\left(x;\alpha,r\right)\text{Gamma}\left(r;\beta,q\right)\mathrm{d}r$. This reveals that the Gamma distribution is a conjugate prior for $\exp(\mu_i)$. We use $p\left(\mu_i\right) = \text{Gamma}\left(\mu_i;1,1\right)$, a Gamma distribution with shape and scale set to 1.

The full conditional of σ does not resemble a known distribution and thus it is sampled with a Metropolis step. As a prior for σ we use a positive half normal distribution, $p\left(\sigma\right) = \mathcal{N}^+\left(0,1\right)$.

B.4 Evaluating Elementary Symmetric Polynomials of High Order

A computational challenge is to evaluate the elementary symmetric polynomials of a high order. Using the recurrence relation (Baker & Harwell, 1996; Fischer, 1974):

$$e_s\left(\boldsymbol{\mu}\right) = e_{s-1}\left(\boldsymbol{\mu}_{-i}\right)\mu_i + e_s\left(\boldsymbol{\mu}_{-i}\right).$$

one can compute all elementary symmetric functions required to compute the normalizing constant of the Curie-Weiss model

$$\sum_{s=0}^{E} e_s\left(\exp(\boldsymbol{\mu})\right)\exp\left(\frac{\sigma}{E}s^2\right)$$

in $\mathcal{O}(E^2)$. However, we can improve on this. A well-known Gaussian integral is

$$\exp\left(\frac{\sigma}{E}s^2\right) = \frac{1}{\sqrt{\pi}}\int_{\mathbb{R}}\exp\left(2\sqrt{\frac{\sigma}{E}}s\eta - \eta^2\right)\ \mathrm{d}\eta$$

Using this we may write the normalizing constant as follows:

$$\begin{aligned}\sum_{s=0}^{E} e_s\left(\exp(\boldsymbol{\mu})\right)\exp\left(\frac{\sigma}{E}s^2\right) &= \sum_{s=0}^{E} e_s\left(\exp(\boldsymbol{\mu})\right)\frac{1}{\sqrt{\pi}}\int_{\mathbb{R}}\exp\left(2\sqrt{\frac{\sigma}{E}}s\eta - \eta^2\right)\ \mathrm{d}\eta\\ &= \frac{1}{\sqrt{\pi}}\int_{\mathbb{R}}\exp\left(-\eta^2\right)\sum_{s=0}^{E} e_s\left(\exp(\boldsymbol{\mu})\right)\exp\left(2\sqrt{\frac{\sigma}{E}}s\eta\right)\ \mathrm{d}\eta\end{aligned}$$

Now we recognize that $\exp\left(2\sqrt{\frac{\sigma}{E}}s\eta\right)$ is a polynomial in s and can use the ordinary generating function of the elementary symmetric polynomials (Egge, 2019, p. 38), that is,

$$\sum_{s=0}^{E} e_s\left(x_1,\ldots,x_E\right)t^s = \prod_{s=1}^{E}(1 + x_s t)$$

to obtain

$$\sum_{s=0}^{E} e_s\left(\exp(\boldsymbol{\mu})\right)\exp\left(\frac{\sigma}{E}s^2\right) = \frac{1}{\sqrt{\pi}}\int_{\mathbb{R}}\exp\left(-\eta^2\right)\left[\prod_{s=1}^{E}\left(1+\exp\left(\mu_s+2\sqrt{\frac{\sigma}{E}}\eta\right)\right)\right]\ \mathrm{d}\eta$$

For large graphs, it is both numerically and computationally more efficient to compute the integral using Gauss-Hermite quadrature than to compute all elementary symmetric functions. This implies we compute the following approximation

$$\begin{aligned}\frac{1}{\sqrt{\pi}}\int_{\mathbb{R}}\exp\left(-\eta^2\right)&\left[\prod_{s=1}^{E}\left(1+\exp\left(\mu_s+\sqrt{\frac{\sigma}{E}}\eta\right)\right)\right]\ \mathrm{d}\eta\\ \approx \frac{1}{\sqrt{\pi}}\sum_{m=1}^{M} w_m &\left[\prod_{s=1}^{E}\left(1+\exp\left(\mu_s+\sqrt{\frac{\sigma}{E}}\eta_m\right)\right)\right]\end{aligned}$$

where w_m are the Gauss-Hermite weights and η_m are the roots of the Hermite polynomials. The weights and roots are efficiently obtained using the Julia package FastGaussQuadrature.jl.

C Structure Recovery of Modular Data

Here we illustrate the capacity of our multilevel approach to recover a modular structure. A potential drawback of the Curie-Weiss model is that the interaction term (σ) treats edges as interchangeable and is only impacted by the number of edges simultaneously present in the individual networks. This simulation shows that differences in modularity are captured by the thresholds ($\boldsymbol{\mu}$), which vary across edges. We used a stochastic block model (SBM) as a group-level structure to simulate data with a modular structure. The SBM implies that each node belongs to a block and that the probability of an edge between two nodes is higher for nodes within the same block than nodes in different blocks. The edge-probabilities were randomly sampled from two normal distributions truncated to [0, 1], $\mathcal{N}\left(1/2, 1/12\right)$ for the probabilities within a block and $\mathcal{N}\left(0, 1/15\right)$ for the probabilities between blocks. We simulated 6 blocks and each block contained between 5 and 7 nodes. Next, we simulated individual-level adjacency matrices using the SBM as the true model. The sample precision matrices were drawn from a G-Wishart distribution with scale matrix S, where S was a correlation matrix with a similar block structure as the SBM. In particular, correlations within a block were drawn from a normal distribution with mean 0.35 and variance 0.05 truncated to [0, 1]. Correlations between a block were drawn from a normal distribution with mean 0 and variance 0.05 truncated to [−1, 1]. Altogether, we simulated 1000 time points for 37 nodes for 100

participants. Figure C.3 shows on the left the true model parameters and on the right the sample characteristics. The block structure is clear in the

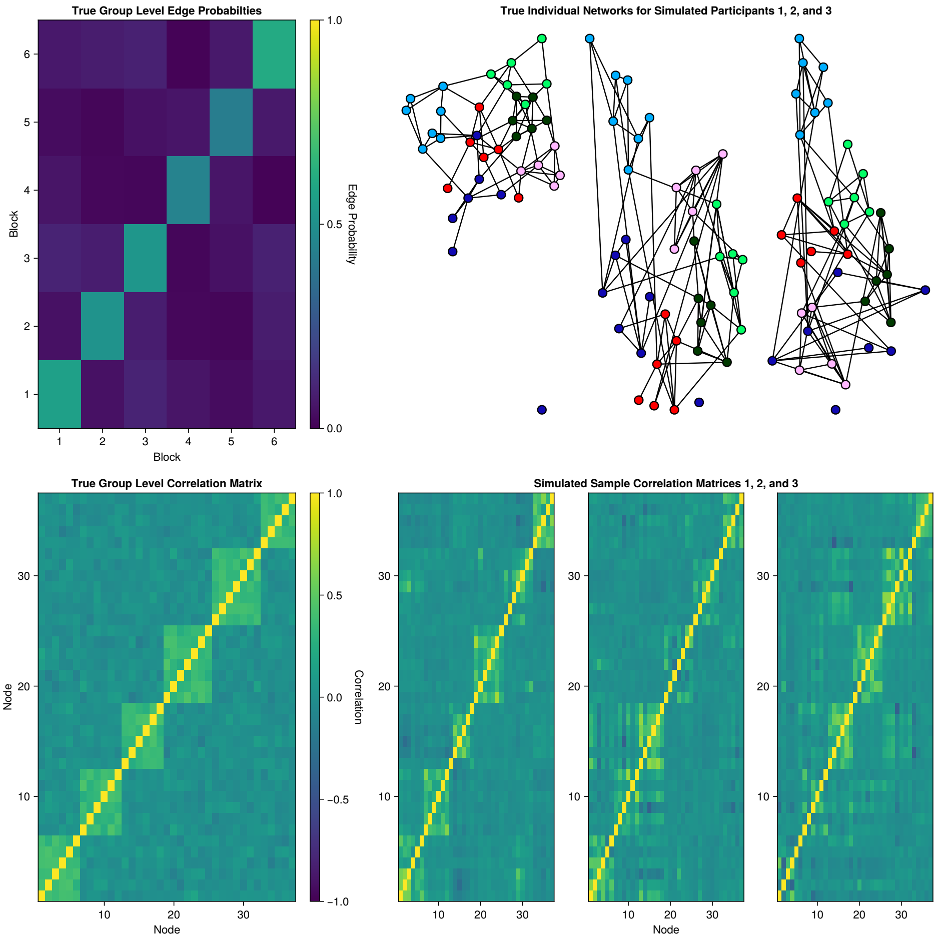

Figure C.3: Group level parameters and sample statistics for three simulated participants.

data generating parameters, but more vague in the sample statistics, and there is considerable variance between the simulated participants.

We analyzed the data with three values for the hyperparameter $v_0 \in \{0.05, 0.1, 0.5\}$, to illustrate how well the modular structure is recovered, and to showcase the impact of this hyperparameter on the results. After the analysis we first examined the $\hat{R}$-statistic for the parameters of the Curie-Weiss models to assess the convergence of the MCMC chains, which is shown in Figure C.4. All $\hat{R}$-statistics are smaller than 1.1, indicating that the MCMC chains converged.

Next we examined the structure recovery for each value of v_0, which is shown in Figure C.5. the block structure is recovered regardless of the value

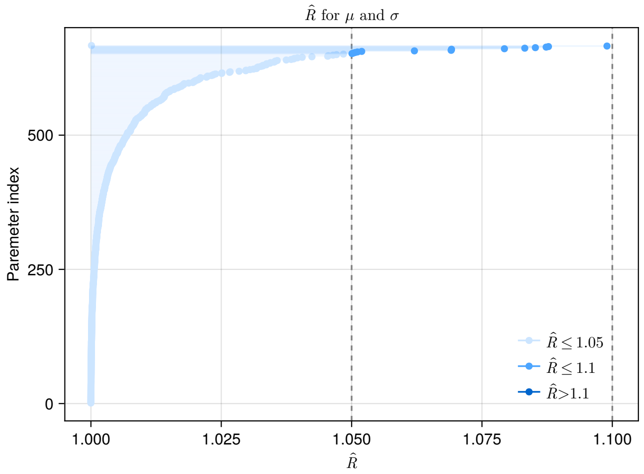

Figure C.4: $\hat{R}$-statistic for the μ and σ parameters of the Curie-Weiss model.

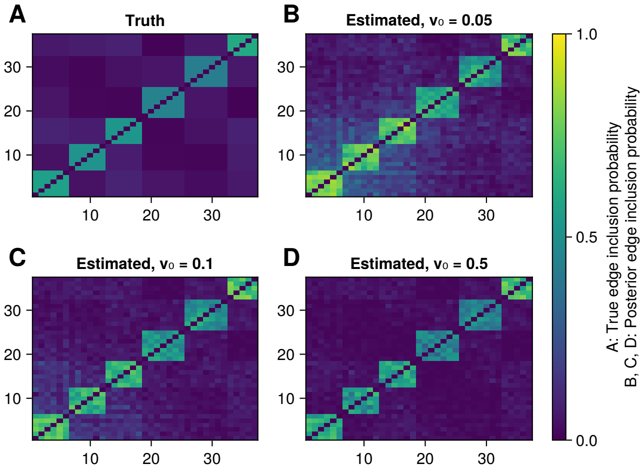

Figure C.5: Recovery of the stochastic block model for various v_0.

of v_0. As v_0 increases the width of the spike increases and so more edges are excluded, leading to a lower inclusion probability. In the results for $v_0 = 0.05$, panel B Figure C.5, there appear to be substantial false positives among the edges between the blocks.

Finally, we examined the parameter recovery of the precision matrix estimates. Figure C.6 shows a similar pattern as in Figure C.5. The smallest value of v_0 leads to increased variance in the parameter estimates, possibly because there is less shrinkage because fewer edges are excluded.

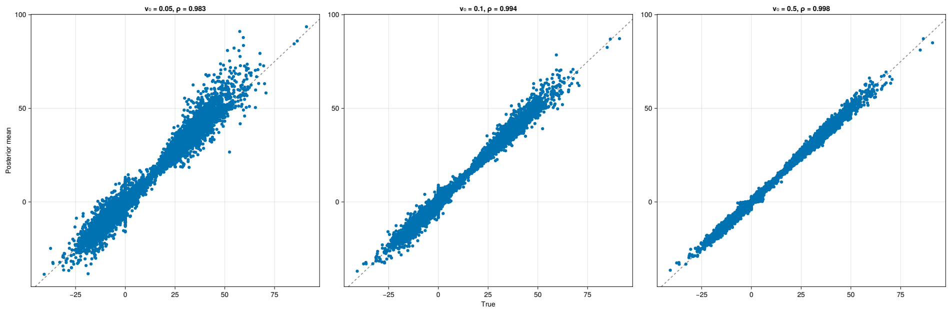

Figure C.6: Recovery of the precision matrix for all 100 simulated participants for various v_0.

D Structure Recovery under Temporal Autocorrelation

A shortcoming of the individual-level model we use is that it assumes there are no temporal relationships. Here we investigate the effect of autocorrelation on the model results. To this end, we simulated data as before but used a matrix normal distribution for the true individual-level networks. The matrix normal distribution generalizes the multivariate normal distribution such that a single realization is a matrix, i.e., all observed time points of a single participant, instead of a vector, i.e., one observed time point of a participant. It is determined by a $P \times T$ mean matrix, a $P \times P$ covariance matrix $\boldsymbol{\Sigma}$, and a $T \times T$ covariance matrix $\boldsymbol{V}$. For simplicity, we assume that the mean matrix is zero. The covariance matrix $\boldsymbol{\Sigma}$ has the same interpretation as before and models the relationships between the nodes. The covariance matrix $\boldsymbol{V}$ describes the relationships between time points, and can model temporal correlations. For example, suppose the data are generated with a lag-1 autocorrelation of .3. Then $\boldsymbol{V}$ is banded so that the off-diagonal elements are the equal ($\boldsymbol{V}_{12} = \boldsymbol{V}_{23} = \boldsymbol{V}_{(i+t)(j+t)}$. The exact values are determined by both the variance and the process used to induce the autocorrelation.

We simulated $T = 250$ time points for $P = 30$ nodes and $N = 75$ participants. The individual-level precision matrices were drawn from a G-Wishart distribution. These varied between participants, but were constant across the simulation runs. We determined $\boldsymbol{V}$ as the expected covariance matrix of an AR process. Specifically, we considered three different levels of autoregression, 1) a high level with $\{0.25, 0.20, \ldots, 0.05\}$, a medium level determined by $\{0.15, 0.10, 0.05\}$, and no autoregression. Figure D.7 illustrates the three levels of autocorrelation. In addition, we varied the overall 'impact' of V relative to the precision matrix $\boldsymbol{\Omega}$. It is necessary to consider some notion of impact, because if the variance of V is large relative to $\boldsymbol{\Omega}$,

then one would expect it to be difficult to accurately retrieve $\mathbf{\Omega}$, as the effect of V to the GGM is simply noise. We quantified the impact as the ratio of the log determinant of V over the log determinant of $\mathbf{\Omega}$, and varied this from $\{.2, .5, 1, 2, 5\}$. In total we simulated 15 datasets. For each dataset we constructed an MCMC chain with 150,000 iterations and discarded the first 25,000 iterations.

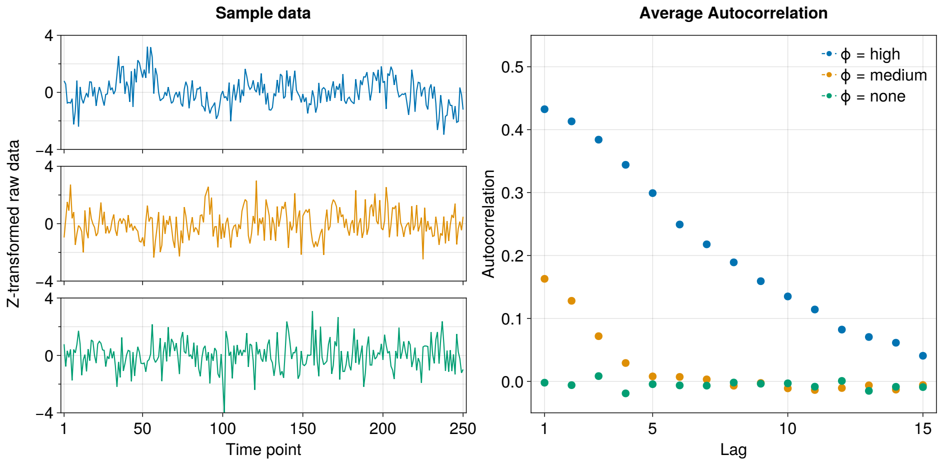

Figure D.7: The left panel shows three sample time series. The right panel shows the sample autocorrelation averaged over the 75 simulated participants.

Figure D.8 shows the recovery of the individual-level precision matrices and the individual-level structure. The left panel shows that the correlation between the true precision matrices and the posterior means is high in all simulation conditions. In the middle panel, however, we see that the root mean squared error $\left(\sqrt{(P^2N)^{-1}\sum_{i,j,k}(\Omega_{ijk}^{\text{true}} - \Omega_{ijk}^{\text{est}})^2}\right)$ increases as the impact of $\boldsymbol{V}$ increases. Similarly, the right panel shows that the structure recovery, as determined by the AUC, also deteriorates as the temporal influence increases. The correlation between the true and estimated precision matrices remains high in all conditions. However, the relationship becomes increasingly slanted, i.e., the slope is positive but not 1, as the temporal impact increases. This implies that the temporal component does not necessarily have a large effect on the estimated direction of a partial correlation, but it does have an effect on the estimated magnitude and structure. Finally, it appears that it is not so much the specific autoregressive process that impacts the recovery, but more so the relative impact of the temporal covariance matrix.

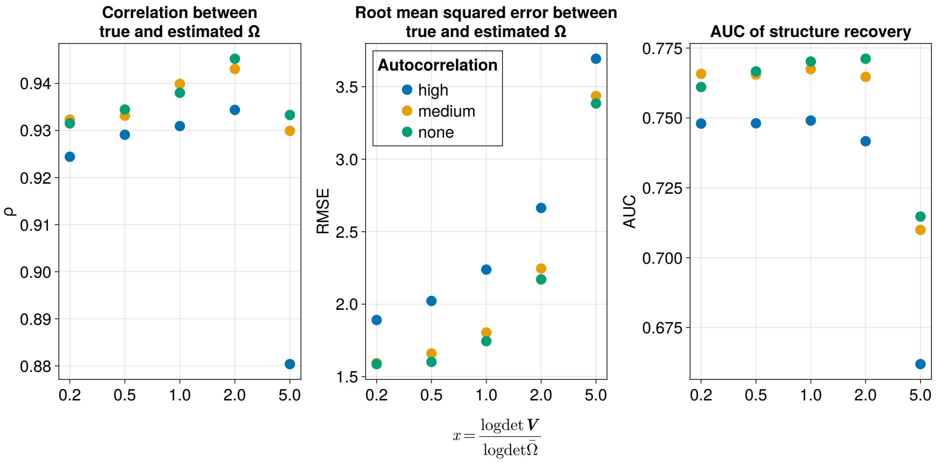

Figure D.8: Parameter and structure recovery for the simulation. As the ratio of the logarithmic determinants (x-axis) becomes greater than 1, the influence of the temporal relations increases and the recovery deteriorates.

References

Baker, F. B., & Harwell, M. R. (1996). Computing elementary symmetric functions and their derivatives: A didactic. *Applied Psychological Measurement*, *20*(2), 169–192.

Busatto, C., & Stingo, F. C. (2023). Inference of multiple high-dimensional networks with the graphical horseshoe prior. *arXiv preprint arXiv:2302.06423*.

Colclough, G. L., Woolrich, M. W., Harrison, S. J., López, P. A. R., Valdes-Sosa, P. A., & Smith, S. M. (2018). Multi-subject hierarchical inverse covariance modelling improves estimation of functional brain networks. *NeuroImage*, *178*, 370–384.

Egge, E. S. (2019). *An introduction to symmetric functions and their combinatorics* (Vol. 91). American Mathematical Soc.

Fischer, G. (1974). Einführung in die theorie psychologischer tests [introduction to the theory of psychological tests]. *Bern: Huber*.

Li, Y., Craig, B. A., & Bhadra, A. (2019). The graphical horseshoe estimator for inverse covariance matrices. *Journal of Computational and Graphical Statistics*, *28*(3), 747–757.

Lingjærde, C., Fairfax, B. P., Richardson, S., & Ruffieux, H. (2022). Scalable multiple network inference with the joint graphical horseshoe. *arXiv preprint arXiv:2206.11820*.

Maris, G., Bechger, T., & Martin, E. S. (2015). A Gibbs sampler for the (extended) marginal Rasch model. *Psychometrika*, *80*, 859–879.

Peterson, C. B., & Stingo, F. C. (2021). Bayesian estimation of single

and multiple graphs. In *Handbook of bayesian variable selection* (pp. 327–348). Chapman and Hall/CRC.

Peterson, C. B., Stingo, F. C., & Vannucci, M. (2015). Bayesian inference of multiple gaussian graphical models. *Journal of the American Statistical Association*, *110*(509), 159–174.

Scott, J. G., & Berger, J. O. (2006). An exploration of aspects of Bayesian multiple testing. *Journal of statistical planning and inference*, *136*(7), 2144–2162.

Tsai, K., Koyejo, O., & Kolar, M. (2022). Joint Gaussian graphical model estimation: A survey. *Wiley Interdisciplinary Reviews: Computational Statistics*, *14*(6), e1582.
